# Effect of reducing saturated fat intake on cardiovascular disease in adults: an umbrella review

**DOI:** 10.3389/fpubh.2024.1396576

**Published:** 2024-06-03

**Authors:** Adolfo Aramburu, Gandy Dolores-Maldonado, Katherine Curi-Quinto, Karen Cueva, Giancarlo Alvarado-Gamarra, Katherine Alcalá-Marcos, Carlos R. Celis, Claudio F. Lanata

**Affiliations:** ^1^Centro de Promoción de Estilos de Vida Saludable, Instituto de Investigación Nutricional (IIN), Lima, Peru; ^2^Faculty of Science Health, Universidad Peruana de Ciencias Aplicadas, Lima, Peru; ^3^Instituto Nacional Cardiovascular “Carlos Alberto Peschiera Carrillo”—INCOR, Lima, Peru; ^4^Department of Pediatrics, School of Medicine, Vanderbilt University, Nashville, TN, United States

**Keywords:** adult, cardiovascular diseases, fatty acids, dietary fats, mortality

## Abstract

**Introduction:**

Our objective was to explore the effect of the reduction of saturated fat (SAF) intake on cardiovascular disease, mortality and other health-related outcomes in adults.

**Methods:**

We conducted an umbrella review, searching Medline, Scopus, EMBASE, Cochrane Library, and LILACS databases for systematic reviews from December 1, 2012, to December 1, 2022. We have included meta-analyses of randomized controlled trials (RCTs) and cohort studies. We extracted effect sizes (95%CI), heterogeneity (*I*^2^), and evidence quality rating based on the population, intervention, comparator, and outcomes.

**Results:**

21 meta-analyses were included (three were from RCTs, and 18 were from cohort studies). Among meta-analyses of RCTs, 15 of the 45 associations were significant. The effect of reduction in SAF intake on combined cardiovascular events (RR 0.79, 95%CI 0.66–0.93) was graded as having moderate certainty of evidence. We found no effect on all-cause mortality, cardiovascular mortality, cancer deaths, and other cardiovascular events. Among meta-analyses of cohort studies, five of the 19 associations were significant. There was an increase in coronary heart disease mortality (HR 1.10, 95% CI 1.01–1.21) and breast cancer mortality (HR 1.51, 95% CI 1.09–2.09) in participants with higher SFA intake compared to reduced SFA. We found no effect on all-cause mortality, cardiovascular mortality, and other cardiovascular events.

**Conclusion:**

This umbrella review found the reduction in SAF intake probably reduces cardiovascular events and other health outcomes. However, it has little or no effect on cardiovascular mortality and mortality from other causes. More high-quality clinical trials with long-term follow-up are needed.

**Systematic review registration**: CRD42022380859.

## 1 Introduction

Cardiovascular disease (CVD) is the leading global cause of death, generating a significant impact on the public health systems of the United States, Europe, and even in low- and middle-income countries (LMCIs), with a secular tendency to increase in recent years. Also, with high annual direct and indirect costs associated with these deaths, including health expenditures and lost productivity (1–5).The American Heart Association (AHA), in conjunction with the National Institutes of Health (NIH) and other government agencies, provides each year a document named the AHA’s *Life’s Essential 8*, which include core health behaviors (smoking, physical activity, diet, and weight) and health factors (cholesterol, blood pressure, and glucose control) that contribute to cardiovascular health (6, 7). In this context, improving the nutritional quality of the diet has been recognized as a relevant lifestyle approach to reducing the risk of atherosclerotic cardiovascular disease (ASCVD) (7, 8).

Public health dietary advice on prevention of CVD has changed over time (9). Regarding saturated fat (SAF) intake, both the American College of Cardiology (ACC) and the AHA (5, 10), as well as the European Society of Cardiology (ESC) (11), recommend replacing saturated with unsaturated fats to reduce the risk of ASCVD. However, these recommendations are based on observational studies and some randomized controlled trials (RCTs) that show discrepancies in their results (9, 12–23). In that sense, certain groups suggest that there is not robust evidence supporting the idea that reducing SAF intake, substituting it with unsaturated fats, or adhering to existing population-wide arbitrary upper limits on SAF consumption will effectively prevent CVD mortality (13, 24–27).

On the other hand, most healthcare interventions evaluated in Cochrane Reviews are not supported by high-quality evidence, and harms are under-reported (28). Additionally, some organizations rely on low-quality evidence to formulate recommendations, justifying their decisions as consensus-based guidelines (29). To ensure recommendations based on high-quality evidence, it is essential to develop trustworthy clinical practice guidelines (CPGs) that are informed by a systematic review of evidence and employ a standardized methodology, such as the Grading of Recommendations Assessment, Development, and Evaluation (GRADE) approach (29, 30).

Therefore, this umbrella review aimed to systematically identify meta-analyses of randomized controlled trials (RCTs) and cohort studies investigating the reduction of saturated fat (SAF) intake and its impact on cardiovascular disease, mortality, and other health-related outcomes in adults.

## 2 Materials and methods

### 2.1 Protocol and registration

This study was performed according to the recommendations of the Preferred Reporting Items for Systematic Reviews and Meta-Analyses (PRISMA) (31). The study protocol was registered in the International Prospective Register of Systematic Reviews (PROSPERO), number CRD42022380859.

### 2.2 Search strategy

We searched Medline, Scopus, EMBASE, Cochrane Library, and LILACS database of systematic reviews from December 01, 2012, to December 1, 2022. No language restriction. Our search strategy included Medical Subject Title (MeSH) terms and free-text terms such as “Saturated Fatty Acid,” “Dietary Fats,” “Cardiovascular Diseases,” “Heart Disease Risk Factors,” and “cardiovascular outcomes.” We adapted the search algorithms to the requirements of each database. The final search strategy is available as Supplementary Table 1.

### 2.3 Eligibility criteria

Studies were included if they met the following criteria: (1) Population: Systematic reviews that include primary studies in adults (over 18 years), at any risk of cardiovascular disease, with or without cardiovascular disease (but not acutely ill), using or not using lipid-lowering medication; (2) Systematic reviews with meta-analyses of RCTs or observational studies (cohort); (3) Intervention/comparator: RCTs comparing reduced SFA intake vs. higher SFA intake, and cohort studies comparing categories of low vs. high SFA intake; and (5) Outcomes: studies that reported cardiovascular events and mortality (all-cause mortality, cardiovascular mortality, and cancer deaths) as primary outcomes, and/or other secondary outcomes such as cancer, diabetes, glucose-insulin homeostasis, lipid profile, body weight, blood pressure, and quality of life. We excluded narrative reviews, scoping reviews, meta-analyses of studies with other study designs, comments, editorials, guidelines, and conference abstracts.

### 2.4 Study selection

Duplicate documents were removed with Endnote X20 software. Six independent authors (AA, GD-M, KC-Q, GA-G, CC, and KA-M) selected the articles by titles and abstracts to identify potentially relevant articles. Then, articles were evaluated in full text to assess their eligibility. Any discrepancies were resolved by discussion with the third reviewer (CFL).

### 2.5 Data extraction

Four independent authors (AA, GA-G, KA-M, and CC) extracted the data. Discrepancies were resolved with consensus. We recorded the following variables: author, year of publication, study design, number of participants and included studies, type of intervention/comparator, outcomes with their effect size with 95% confidence interval (CI), heterogeneity (I^2^), study follow-up range, and GRADE rating. Another author (GA-G) checked the quality of the data before analysis.

We assessed the overlapping of studies according to the “corrected covered area (CCA)’” for each outcome. CCA >5% was considered as significant overlap (32). In this case, the study result with the highest score was prioritized in a score based on the date of publication, methodological quality, and number of primary studies included.

### 2.6 Quality assessment

Four independent authors (AA, GA-G, CC, and KA-M) independently assessed the quality of the included studies using “A Measurement Tool to Assess Systematic Reviews” (AMSTAR-2), a third author (CFL) settled in case of doubt. This tool consists of 16 items (maximum score: 16. and minimum score: 0). Based on the critical domains, we consider high, moderate, low, and critically low quality in the results (33).

### 2.7 Statistical analysis

We have developed a narrative summary of the data from each included systematic review, including effect estimates with their 95% CI, statistical assessment of heterogeneity (*I*^2^), GRADE score (certainty of evidence), and other study characteristics exactly as reported in the included systematic reviews. We performed sensitivity analyses to assess the effect on the outcomes excluded by overlap.

## 3 Results

### 3.1 Study selection

**Figure 1:**
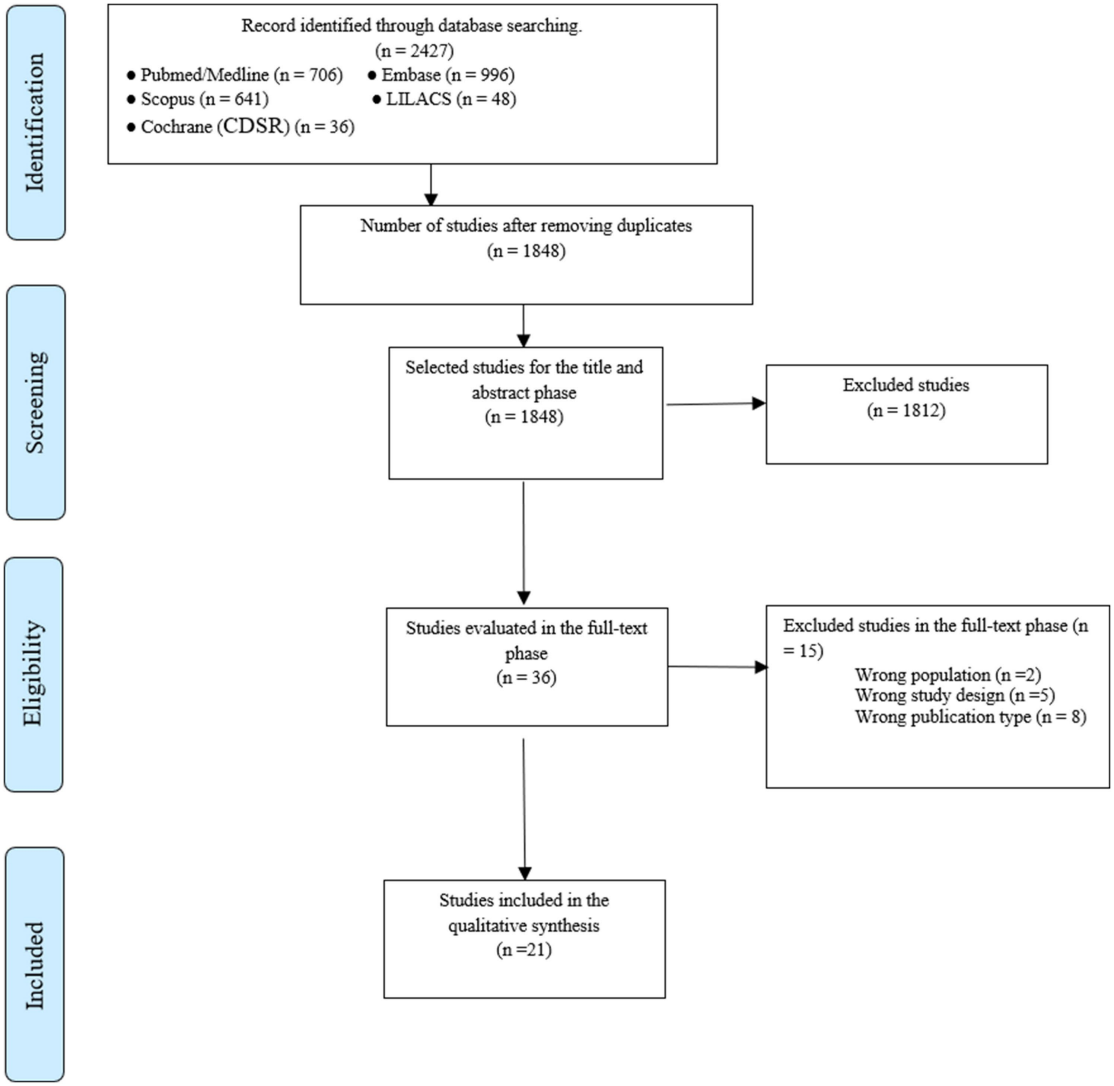
Flow chart of study selection.

A total of 2,427 documents were identified, and 579 duplicates were removed. In the review by title and abstract, there were 1848 potentially eligible studies. Then, 15 documents were excluded during the full-text evaluation (justification available in Supplementary Table 2), and finally 21 meta-analyses were included in the study (Figure 1), three articles were meta-analyses of RCTs (9, 34, 35) and 18 were meta-analyses of cohort studies (17–19, 36–50). After the selection criteria, no studies were excluded due to overlap, but some outcomes were not analyzed because of data overlapping (list of excluded outcomes in Supplementary Table 3).

### 3.2 Characteristics of included studies

The three meta-analyses of RCTs (9, 34, 35) described 45 potential associations of cardiovascular disease and mortality associated with reduction in SAF intake. The number of RCTs were 125, with a sample of 663–56,000 participants and a follow-up duration ranging from 3 to 9 years (additional characteristics in Table 1).

**Table 1:** Characteristics of meta-analyses of randomized clinical trials studying saturated fat intake.

Author (year)	Population	Type of intervention	Comparator	Study follow-up range	No. of included studies	Total participants	Outcomes ^a^	AMSTAR-2 rating
Hooper et al. (9)	Adults (18 years or older, no upper age limit) at any risk of cardiovascular disease, with or without existing cardiovascular disease, using or not using lipid-lower in medication. Participants could be of either gender, but we excluded those who were acutely ill, pregnant or lactating.	Reduced SFA intake^b^	Higher SFA intake^c^	1.5–9 years^d^	15	56,000	1,2,3,4,5,6,7,8,9,10,11,12,13,14,15	High
Imamura et al. (34)	Adults (≥18 years), non-pregnant.	Replacement SFA with MUFA or PUFA	Intake without replacement SFA	3–168 days	102	4,220	11	Critically low
Hannon et al. (35)	Healthy adults (≥18 years) with criteria for overweight and obesity without diagnosis of metabolic disease.	Replacement of SFA with MUFA or PUFA	Intake without replacement in SFA	4–28 weeks	8	663	12	Critically low

No, Number; AMSTAR-2, A Measurement Tool to Assess Systematic Reviews; SFA, Saturated fatty acids; MUFA, Monounsaturated fat; PUFA, Polyunsaturated fat. ^a^1 = All-cause mortality, 2 = Cardiovascular mortality, 3 = Coronary heart disease mortality, 4 = Cancer deaths, 5 = Combined cardiovascular events, 6 = Myocardial infarction, 7 = Non-Fatal myocardial infarction, 8 = Coronary heart disease events, 9 = Stroke, 10 = Cancer diagnoses, 11 = Glucose-insulin homeostasis, 12 = Lipidic profile, 13 = Body weight, 14 = Blood Pressure, and 15 = Quality of life. ^b^By suggesting appropriate nutrient based or food-based aims, or which provided a general dietary aim, such as improving heart health or reducing total fat. The intervention had to be dietary advice, supplementation of fats, oils or modified or low-fat foods, or a provided diet. ^c^Which could be a diet high in saturated fat, or a usual diet (not modified in SFA). ^d^Range of mean years in trial.

The 18 meta-analyses of cohort studies (17–19, 36–50) described 19 potential associations of cardiovascular disease and mortality associated with reduction in SAF intake. The median number of studies per meta-analysis was 11 (interquartile range, IQR, 7–15), the follow-up duration ranged from 1 to 32 years, and the median sample was 462,268 participants (IQR, 318,747-836,322.5 participants) per meta-analysis (additional characteristics in Supplementary Table 4).

### 3.3 Quality of studies

About meta-analyses of RCTs, the quality assessment revealed that one was rated as high quality (score: 16) (9), while two were assessed as critically low quality (score: 13 and 10) (34, 35). About meta-analyses of cohort studies (17–19, 36–50), analysis revealed that three meta-analyses (16.7%) were of low quality, and 15 meta-analyses (83.3%) were of critically low quality, with a median score of 10.5 (interquartile range: 8–12) (details in Supplementary Table 5).

### 3.4 Description and summary of associations

#### 3.4.1 Meta-analyses of RCTs

Fifteen of the 45 associations (33.3%) were statistically significant (*p* < 0.05) based on random-effects models. The identified associations comprised two types of intervention/comparator, including reduced SFA intake vs. higher SFA intake (51.1%) or replacement of SFA with monounsaturated fatty acids (MUFAs) or polyunsaturated fatty acids (PUFAs) vs. intake without replacement in SFA (48.9%). 30 associations analyzed non-repeated outcomes, including nine (30%) cardiovascular and mortality events, six (20%) lipid profile outcomes, nine (30%) glucose-insulin homeostasis outcomes, two (6.7%) blood pressure outcomes, two (6.7%) body weight outcomes, one outcome (3.3%) of quality of life, and another (3.3%) with a diagnosis of cancer. Also, 10 of the 24 associations (41.7%) had heterogeneity (*I*^2^) > 50%, and eight (17.8%) of the 45 associations assessed the certainty of evidence using GRADE (three associations were supported by moderate certainty of evidence, others three were supported by very low, and two associations by low certainty of evidence). Summary of all associations in Table 2 and Supplementary Table 6.

**Table 2:** Summary of primary findings of meta-analyses of randomized clinical trials studying saturated fat intake.

Author (year)	Outcomes	Population	Type of intervention	Comparator	Study follow-up range ^a^	No. of included studies	Intervention/comparator	Measures of effect	Effect size (95%CI)	*I*^2^,%	AMSTA-2 rating	GRADE rating
Mortality												
Hooper et al. (9)	All-cause mortality	Adults (≥ 18 years)^b^	Reduced SFA intake^c^	Higher SFA intake^d^	1.5–8.6	12	1495 en 22819/2053 en 33039	RR	0.96 (0.90, 1.03)	2	High	Moderate
Hooper et al. (9)	Cardiovascular mortality	Adults (≥ 18 years)^b^	Reduced SFA intake^c^	Higher SFA intake^d^	1.5–8.6	11	483 en 21844/613 en 31577	RR	0.94 (0.78, 1.13)	36	High	Moderate
Hooper et al. (9)	Coronary heart disease mortality	Adults (≥ 18 years)^b^	Reduced SFA intake^c^	Higher SFA intake^d^	1.5–8.6	9	415 en 21714/512 en 31445	RR	0.97 (0.82, 1.16)	28	High	Low
Hooper et al. (9)	Cancer deaths	Adults (≥ 18 years)^b^	Reduced SFA intake^c^	Higher SFA intake^d^	1.9–8.6	5	987 en 21270/1485 en 31013	RR	1.00 (0.61, 1.64)	49	High	NR
Cardiovascular events												
Hooper et al. (9)	Combined cardiovascular events^e^	Adults (≥ 18 years)^b^	Reduced SFA intake^c^	Higher SFA intake^d^	1.5–8.6	11	1816 en 21791/2660 en 31509	RR	0.79 (0.66, 0.93)	65	High	Moderate
Hooper et al. (9)	Myocardial infarction	Adults (≥ 18 years)^b^	Reduced SFA intake^c^	Higher SFA intake^d^	1.5–8.6	11	717 en 21725/997 en 31442	RR	0.90 (0.80, 1.01)	10	High	Very low
Hooper et al. (9)	Non-Fatal myocardial infarction	Adults (≥ 18 years)^b^	Reduced SFA intake^c^	Higher SFA intake^d^	1.5–8.6	8	571 en 21559/814 en 31275	RR	0.97 (0.87, 1.07)	0	High	Low
Hooper et al. (9)	Coronary heart disease events	Adults (≥ 18 years)^b^	Reduced SFA intake^c^	Higher SFA intake^d^	1.5–8.6	11	936 en 21743/1325 en 31456	RR	0.83 (0.68, 1.01)	62	High	Very low
Stroke												
Hooper et al. (9)	Stroke	Adults (≥ 18 years)^b^	Reduced SFA intake^c^	Higher SFA intake^d^	1.9–8.6	7	454 en 20602/664 en 30350	RR	0.92 (0.68, 1.25)	9	High	Very low

No, Number; CI, Confidence intervals; *I*^2^, Statistic assessment of heterogeneity; %, Percentage; AMSTAR-2, A Measurement Tool to Assess Systematic Reviews; GRADE, Grading of recommendations, assessment, development, and evaluations; SFA, Saturated fatty acids; RR, Relative risk. NR, Not reported. ^a^Range of mean years in trial. ^b^Adults (18 years or older, no upper age limit) at any risk of cardiovascular disease, with or without existing cardiovascular disease, using or not using lipid-lower in medication. Participants could be of either gender, but we excluded those who were acutely ill, pregnant or lactating. ^c^By suggesting appropriate nutrient based or food-based aims, or which provided a general dietary aim, such as improving heart health or reducing total fat. The intervention had to be dietary advice, supplementation of fats, oils or modified or low-fat foods, or a provided diet. ^d^Which could be a diet high in saturated fat, or a usual diet (not modified in SFA). ^e^These included people experiencing any of the following: cardiovascular death, cardiovascular morbidity (non-fatal myocardial infarction, angina, stroke, heart failure, peripheral vascular events, and atrial fibrillation) and unplanned cardiovascular interventions (coronary artery bypass surgery or angioplasty).

#### 3.4.2 Meta-analyses of cohort studies

Five of the 19 associations (26.3%) were statistically significant (*p* < 0.05) based on random-effects models. The identified associations comprised one type of intervention/comparator: higher vs. reduced SFA intake. 18 associations analyzed non-repeated outcomes, including 12 (66.7%) cardiovascular and mortality events, five (27.8%) with a diagnosis of cancer, and one (5.5%) with a diagnosis of diabetes. Additionally, seven of the 17 associations (41.2%) had heterogeneity (*I*^2^) > 50%, and two (10.5%) of the 19 associations assessed the strength of evidence using GRADE (both were supported by very low certainty). Summary of all associations in Table 3 and Supplementary Table 7.

**Table 3:** Summary of primary findings of meta-analyses of cohorts studying saturated fat intake.

Author (year)	Outcomes	Population	Type of exposure	Comparator	Study follow-up range (years)	No. of included studies	Intervention/comparator	Measures of effect	Effect size (95%CI)	*I*^2^,%	AMSTA-2 rating	GRADE rating
Mortality												
Mazidi et al. (17)	All-cause mortality	Adults (>18 years)	Higher SFA intake: According to percentiles, gr/day, % energy, increase in different units	Reduced SFA intake: According to percentiles, gr/day, % energy, increase in different units	3.7–32	14	NR	HR	1.05 (0.99, 1.12)	40	Critically low	NR
Kim et al. (18)	Cardiovascular disease mortality	Adults (>20 years) without pre-existing disease at baseline	Higher SFA intake:	Reduced SFA intake:	6.1–32	10	NR	RR	1.02 (0.92, 1.12)	78.2	Critically low	NR
			Higher intake category	Lowest intake category								
			g/day (34.7)	g/day (67.5)								
			% total energy (range: 2.5–8.7%)	% total energy (range: 7.3–17.9%)								
Mazidi et al. (17)	Coronary heart disease mortality	Adults (>18 years)	Higher SFA intake: According to percentiles, gr/day, % energy, increase in different units	Reduced SFA intake: According to percentiles, gr/day, % energy, increase in different units	4.5–23	14 ^a^	NR	HR	1.10 (1.01, 1.21)	NR	Critically low	NR
Cheng et al. (49)	Fatal stroke	Adults (20–89 years)	Higher SFA intake: Higher intake category (range: 20.3–21 gr/day)	Reduced SFA intake: Lowest intake category (range: 7–9.4 gr/day)	10.6–23	4^b^	NR	RR	0.75 (0.59, 0.94)	0	Critically low	NR
Kim et al. (18)	Cancer mortality	Adults (>20 years) without pre-existing disease at baseline	Higher SFA intake:	Reduced SFA intake:	6.1–32	6	NR	RR	1.09 (1.00, 1.18)	73.2	Critically low	NR
			Higher intake category	Lowest intake category								
			% total energy (range: 3–8.7%)	% total energy (range: 7.3–17.9%)								
Brennan et al. (50)	Breast cancer mortality	Adults (19–75 years)	Higher SFA intake: Higher intake category	Reduced SFA intake: Lowest intake category	5.5–18	4 ^c^	NR	HR	1.51 (1.09, 2.09)	15	Critically low	NR
Cardiovascular events												
Zhu et al. (19)	Cardiovascular disease	Adults (>18 years)	Higher SFA intake: Higher intake category	Reduced SFA intake: Lowest intake category	NR	56	NR	HR	0.97 (0.93, 1.02)	56.8	Critically low	NR
de Souza et al. (38)	Coronary heart disease	Adults (>16 years)	Higher SFA intake:	Reduced SFA intake:	1–20	12	6383 en 267,416/NR	RR	1.06 (0.95, 1.17)	47	Low	Very low
			Higher intake category	Lowest intake category								
			g/day (range: 7–34.7)	g/day (range: 21–67.5)								
			% total energy (range: 0.7–22.3%)	% total energy (range: 1.5–36.2%)								
Chowdhury et al. (48)	Coronary disease	Adults (>18 years) from general populations or with estable cardiovascular disease	Higher SFA intake: Top third of baseline intake	Higher SFA intake: Bottom third of baseline intake	1.3–30.7	20	NR	RR	1.02 (0.97, 1.07)	NR	Critically low	NR
Stroke												
Cheng et al. (49)	Stroke	Adults (20–89 years)	Higher SFA intake: Higher intake category (range: 15.4–36 gr/day)	Reduced SFA intake: Lowest intake category (range: 7–20 gr/day)	7.6–23	15^d^	NR	RR	0.89 (0.82, 0.96)	37.4	Critically low	NR
de Souza et al. (38)	Ischemic stroke	Adults (>18 years)	Higher SFA intake:	Reduced SFA intake:	7.6–32	12	6226 en 339,090/NR	RR	1.02 (0.90, 1.15)	59	Low	Very low
			Higher intake category	Lowest intake category								
			g/day (range: 7–55.7)	g/day (range: 15.4 to 86.6)								
			% total energy (range: 0.7–36.1%)	% total energy (range: 1.5– 44.8%)								
Muto and Ezaki (41)	Intracerebral hemorrhage	Adults (34–89 years)	Higher SFA intake: Higher intake category	Reduced SFA intake: Lowest intake category	10.4–14	5	NR	HR	0.69 (0.48,1.00)	58.1	Critically low	NR
Kang et al. (45)	Subarachnoid hemorrhage	Adults (>18 years)	Higher SFA intake: Higher intake category (range: 15.4–50.4 gr/day)	Reduced SFA intake: Lowest intake category (range: 5.2–26.8 gr/day)	11.1–14.1	3	NR	RR	0.97 (0.69, 1.37)	0	Low	NR

No, Number; CI, Confidence intervals; *I*^2^, Statistic assessment of heterogeneity; %, Percentage; AMSTAR-2, A Measurement Tool to Assess Systematic Reviews; GRADE, Grading of recommendations, assessment, development, and evaluations; SFA, Saturated fatty acids; gr, Gramos; NR, No reported; HR, Hazard ratio; RR, Relative risk. ^a^Adjustment for confounding variables: Age, race, sex, education, marital status, poverty to income ratio, physical activity, SBP, smoking, serum cholesterol, alcohol, BMI, ancestry, left ventricular hypertrophy, presence of initial malignant disease, energy intake, serum lipids, systolic blood pressure, glucose intolerance, energy, physical activity, history of hypertension, family history of myocardial infarction < 60 years, profession, dietary fiber, social class, education, frequency of exercise, dietary supplements, diabetes, HDL, LDL, triacylglycerol, hypertension, examination year, % energy from protein, other fatty acids, years of schooling, and waist to hip ratio. ^b^Adjustment for confounding variables: Age, sex, blood pressure, total cholesterol, smoking, diabetes, hypertension, coronary heart disease, Quetelet index, radiation dose, alcohol, mental stress, physical activity, walking, sports, education, total energy intake, animal protein, polyunsaturated fatty acids, vegetables, and fruits. ^c^Adjustment for confounding variables: Age at menarche, Quetelet index, total energy intake, age at diagnosis, smoking, body weight, dietary factors, physical activity, BMI, weight change, reproductive factors, treatment, and breast cancer stage at diagnosis. ^d^Adjustment for confounding variables: Age, sex, race, marital status, income, socio-economic status, education, Quetelet index, mental stress, alcohol, smoking, physical activity, walking, sports, menopausal status, hormone use, aspirin use, other medications, multivitamins, radiation dose, blood pressure, total cholesterol, diabetes, hypertension, coronary heart disease, left ventricular hypertrophy, atrial fibrillation, family history of myocardial infarction, total energy intake, fiber, potassium, vitamin E, C and calcium, animal protein, polyunsaturated fatty acids, vegetables, and fruits.

### 3.5 Findings of outcomes

#### 3.5.1 Meta-analyses of RCTs

**Figure 2:**
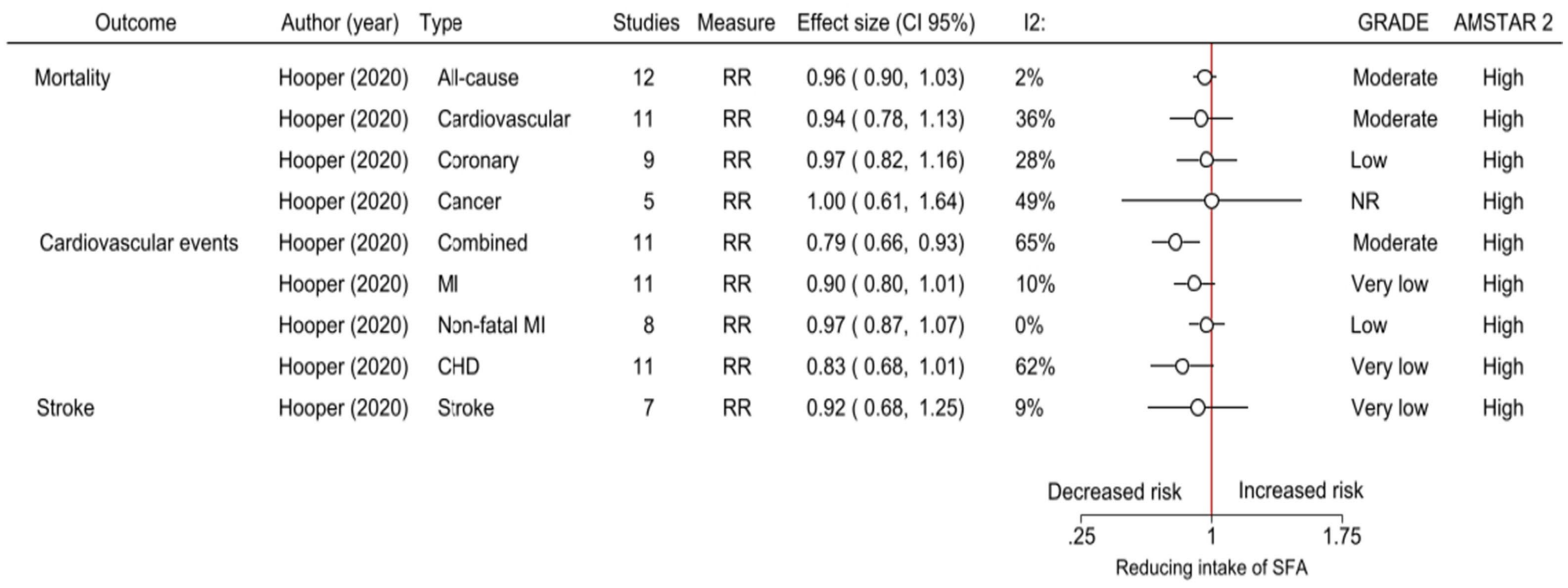
The effects of reduced intake of saturated fats as reported in meta-analyses of RCTs. CI, Confidence intervals; *I*^2^, Statistic assessment of heterogeneity; GRADE, Grading of recommendations, assessment, development, and evaluations; AMSTAR-2, A Measurement Tool to Assess Systematic Reviews; RR, Relative risk; MI, Myocardial infarction; CHD, Coronary heart disease; NR, No reported; and SFA, Saturated fatty acids.

There was a 21% reduction in combined cardiovascular events in people who had reduced SFA compared with those on higher SFA intake (RR 0.79, 95%CI 0.66–0.93, *I*^2^ = 65%, 11 RCTs) (moderate certainty of evidence, GRADE) (9). We found no effect on all-cause mortality, cardiovascular mortality, cancer deaths, and other cardiovascular events such as myocardial infarction, coronary heart disease events, and stroke (moderate, low, and very low certainty of evidence, GRADE) (summary of the studies in Table 2 and Figure 2; and details of the GRADE assessment in Supplementary Table 8).

About secondary outcomes, the certainty of evidence by GRADE was not reported. There was a reduction in total cholesterol (mean difference, MD, −0.24 mmol/L, 95% CI −0.36 to −0.13, *I*^2^ = 60%, 13 RCTs) and low-density lipoprotein cholesterol (LDL-C) (MD −0.19 mmol/L, 95% CI −0.33 to −0.05, *I*^2^ = 37%, five RCTs) in participants with reduced SFA compared to higher SFA (high quality, AMSTAR-2) (9). Also, there was a reduction in body weight (MD −1.77 kg, 95% CI −3.54 to −0.01, *I*^2^ = 77%, six RCTs), and body mass index (BMI) (MD −0.42 kg/m^2^, 95% CI −0.72 to −0.12, *I*^2^ = 62%, six RCTs) (high quality, AMSTAR-2) (9). Regarding the glucose-insulin homeostasis, there was a reduction in glucose tolerance test (GTT) after reducing SFA intakes compared to higher SFA (high quality, AMSTAR-2) (9). Replacing SFA with PUFAs or MUFAs lowered fasting glucose, hemoglobin A1c (HbA1c), C-peptide, and homeostatic model assessment of insulin resistance (HOMA-IR) (34). Furthermore, it enhanced insulin secretion capacity (based on acute insulin response) and increased fasting insulin levels (critically low quality, AMSTAR-2) (34). Only one RCT reported assessing quality of life, they found a small improvement in the group with lower SFA intake (high quality, AMSTAR-2) (9). Summary of all significant and nonsignificant associations in Table 2 and Supplementary Table 6.

#### 3.5.2 Meta-analyses of cohort studies

The certainty of evidence evaluated by GRADE was not documented for certain outcomes. There was an increase in coronary heart disease mortality (HR 1.10, 95% CI 1.01–1.21, *I*^2^ = not reported, 14 cohort studies) (low quality, AMSTAR-2) (17) and breast cancer mortality (HR 1.51, 95% CI 1.09–2.09, *I*^2^ = 15%, four cohort studies) (critically low quality, AMSTAR-2) (50) in participants with higher SFA intake compared to reduce SFA (Table 3).

Among the two associations supported by very low certainty of evidence (GRADE) (38), we found no effect on coronary heart disease (follow-up range: 1–20 years) and ischemic stroke (follow-up range: 7.6–32 years) in participants with higher SFA intake compared to reduced SFA (details of the GRADE assessment in Supplementary Table 9). We also found no effect on all-cause mortality, cardiovascular disease mortality, cancer mortality, and others cardiovascular events as cardiovascular disease, intracerebral hemorrhage, and subarachnoid hemorrhage, with a follow-up range of 1.3–32 years (low and critically low quality, AMSTAR-2) (Table 3).

**Figure 3:**
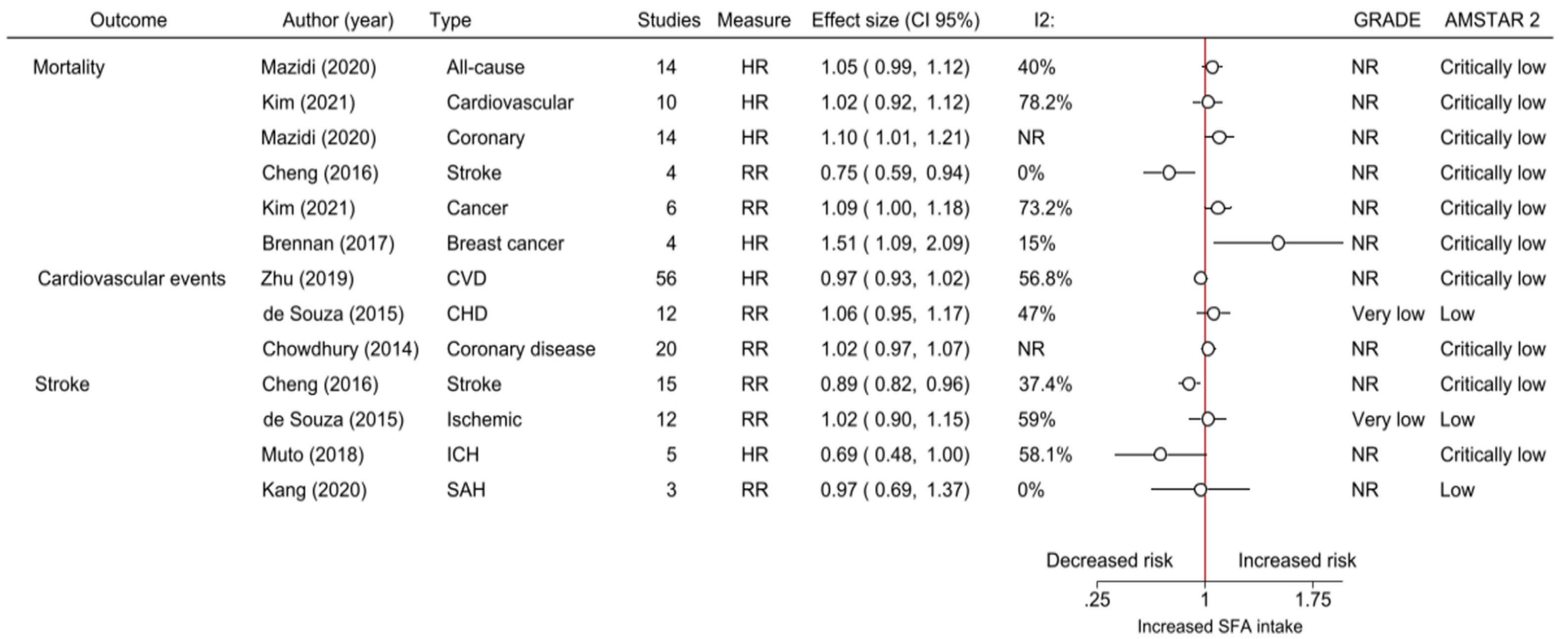
The effects of increased intake of saturated fats as reported in cohort meta-analyses. CI, Confidence intervals; *I*^2^, Statistic assessment of heterogeneity; GRADE, Grading of recommendations, assessment, development, and evaluations; AMSTAR-2, A Measurement Tool to Assess Systematic Reviews; HR, Hazard ratio; RR, Relative risk; CVD, Cardiovascular disease; CHD, Coronary heart disease; ICH, Intracerebral hemorrhage; SAH, Subarachnoid hemorrhage; NR, No reported; and SFA, Saturated fatty acids.

On the other hand, there was a reduction in fatal stroke (RR 0.75, 95% CI 0.59–0.94, *I*^2^ = 0, 4 cohort studies) (critically low quality, AMSTAR-2) (49) and stroke (RR 0.89, 95% CI 0.82–0.96, *I*^2^ = 37.4, 15 cohort studies) (critically low quality, AMSTAR-2) (49) in participants with higher SFA intake compared to reduce SFA (summary of the primary outcomes in Table 3 and Figure 3).

About secondary outcomes, there was an increase in liver cancer (RR 1.34, 95% CI 1.06–1.69, *I*^2^ = 16.9, 5 cohort studies) (critically low quality, AMSTAR-2) (36) in participants with higher SFA intake compared to reduce SFA (Supplementary Table 7).

In all significant outcomes, adjustment for confounding variables was performed. Summary of all significant and nonsignificant associations in Table 3 and Supplementary Table 7.

### 3.6 Sensitivity analyses

We have conducted sensitivity analyses, taking into account the potential impact of excluding outcomes due to overlap in cohort studies. We found similar results about mortality (there is no effect on all-cause mortality and cardiovascular disease mortality). In the context of stroke, participants with higher SFA intake experienced a reduction in stroke events compared to those with lower SFA intake. However, differences in the results were observed. While there is no effect on stroke mortality, there was a reduction in events related to stroke subtypes (ischemic and hemorrhagic) (Supplementary Table 10).

## 4 Discussion

Our findings indicate that the effect of reduction in SAF intake probably reduces cardiovascular events and other health outcomes. However, it has little or no effect on cardiovascular mortality and mortality from other causes. Additionally, we observed a reduction in lipid profile (total cholesterol and LDL-C), body weight, BMI, and an improvement in glucose-insulin homeostasis. Moreover, it could enhance the quality of life and reduce the risk of liver cancer. Finally, participants with higher SFA intake, compared to those with reduced SFA intake, may experience a decrease in fatal stroke and stroke events, as suggested by some observational studies.

In our study, we did not observe differences in mortality in RCTs, including both cardiovascular and other causes. However, we found a little effect in observational studies with wide in confidence intervals, such as Cheng et al. (fatal stroke) (49) and Brennan et al. (breast cancer mortality) (50). This suggests the mortality could occur with considerable variability in other countries or contexts. This could be due to the infrequency of the outcome and the small number of studies included, despite having a large study sample. Furthermore, discrepancies among studies could be attributable to the different biological effects produced by various types of saturated fatty acids, influenced by factors such as the food matrix and dietary carbohydrate content. Individual and methodology factors, including age, sex, adiposity levels, and the shorter follow-up time in RCTs, may also contribute to these variations (24–26, 51, 52). Consistent with our findings, the current recommendations from ACC, AHA, and ESC do not justify their decisions based on mortality results but instead aim to reduce the risk of ASCVD (5, 10, 11).

Regarding the risk of ASCVD, the CPGs recommendations are derived from a combination of observational studies and data from RCTs (5, 10, 11). However, our findings reveal heterogeneity results. In RCTs, we found significant differences observed when meta-analyzed and when creating a composite outcome that groups various types of cardiovascular events, without differences when meta-analyzed by outcome (9). ACC, AHA, and ESC (5, 10, 11) have taken a conservative approach and decided to recommend replacing SAF by PUFAs, principally. This decision was likely made, emphasizing that even a small percentage reduction in cardiovascular-related health outcomes can substantially decrease the number of people developing CVD, both nationally and globally, along with the associated healthcare costs (5). On the other hand, we observed a reduction in lipid profile, body weight, BMI, and an improvement in glucose-insulin homeostasis. This is compatible with the majority of published data about that (9, 34, 53, 54), and it could be another reason to justify the reduction of SAF intake. Additionally, it could enhance the quality of life and reduce risk of liver cancer, but it is necessary to have more studies to confirm it. Based on these issues, rather than having an universal recommendation, practitioners should give personalized recommendations, taking into account factors such as the habitual dietary patterns of individuals, nutritional status, income level, comorbidities, physical activity, and country-level nutrition data.

It is important to avoid recommendations based on low or very low-quality studies. This is crucial to prevent discordant recommendations, avoid harm to patient care, discourage future RCTs, minimize confusion and frustration among practitioners, and manage health system resources effectively, especially in LMCIs (30, 55). Furthermore, we should assess the benefits of interventions based on critical and important outcomes, avoiding reliance on surrogate measures (56).

In relation to stroke, observational studies suggest a decrease in both fatal stroke and overall stroke events with higher SFA intake. However, there is significant variability among the other studies (13, 17, 41, 45, 49), indicating a complex relationship and highlighting the need for further research to fully understand the underlying mechanisms. Another interesting and dual behavior can be observed with high-density lipoprotein cholesterol (HDL-C) levels. Classically, it is known that HDL-C is inversely associated with CVD risk (11). However, some studies report that very high levels of HDL-C may increase CVD risk and mortality (57, 58). Further clarification is needed in future studies.

### 4.1 Limitations and strengths

This study has limitations that are important to mention. First of all, due to the study design (umbrella review, where the unit of searching and data analysis is the systematic review rather than the primary study) (59), our intention was to provide a broad overview of the impact of SAF intake on cardiovascular disease. Our aim was not to evaluate this effect on an individual level, nor did we intend to assess all primary studies included in each meta-analysis. Instead, our focus has been on analyzing the methodology and findings of each systematic review, while acknowledging the inherent limitations in this approach. Secondly, despite conducting a systematic review, we were unable to make recommendations comparable to CPGs. High-quality evidence is the cornerstone of assessing the benefits and harms of an intervention. To maximize the trustworthiness of recommendations within the context of CPGs, they should be rigorously and transparently developed using a standardized methodology. This process should take into account expert opinions, as well as considerations of equity, resource utilization, acceptability, and feasibility (59). Third, we could not re-analyze the outcome data of the systematic reviews, as it was not an objective in our study protocol. Instead, we presented the outcome data exactly as they appear in the included systematic reviews. We believe that this overview format is the most appropriate and a feasible way to address our research question. Fourth, a minority of authors reported evaluations of the certainty of the evidence using the GRADE approach (17.8 and 10.5% of associations in meta-analyses of RCTs and observational studies). However, we assessed the quality of all systematic reviews included using the AMSTAR-2 tool. Finally, the maximum follow-up duration reported in RCTs was 9 years. To address concerns about the potential lack of time to obtain mortality outcomes, it may be necessary for RCTs to have a longer duration. On the other hand, systematic reviews of observational studies reported a maximum follow-up of 32 years. While differences in mortality were found in observational studies, these studies showed a small effect, imprecision, heterogeneity, and a high risk of bias. For all these reasons, our results are exploratory, and should be interpreted with caution.

The strength of this study includes a systematic and exhaustive search of the literature, inclusion of a large body of evidence, and the incorporation of systematic reviews of both RCTs and observational studies. Also, this study stands out as the first umbrella review that focuses on SAF intake’s impact on cardiovascular outcomes, considering data from both RCTs and observational studies.

## 5 Conclusion

This umbrella review found the reduction in SAF intake probably reduces cardiovascular events and other health outcomes. However, it has little or no effect on cardiovascular mortality and mortality from other causes. A healthy diet and physical activity remain the cornerstones of CVD prevention in all individuals. However, recommendations should be individualized considering factors such as nutritional status, comorbidities, and income level. Additionally, high-quality clinical trials with long-term follow-up are needed to investigate the effects of reduced SAF intake on cardiovascular-related health outcomes.

## Data availability statement

The original contributions presented in the study are included in the article/Supplementary material; further inquiries can be directed to the corresponding authors.

## Author contributions

AA: Writing – original draft, Writing – review & editing. GD-M: Writing – original draft, Writing – review & editing. KC-Q: Writing – original draft, Writing – review & editing. KC: Writing – original draft, Writing – review & editing. GA-G: Writing – original draft, Writing – review & editing. KA-M: Writing – original draft, Writing – review & editing. CC: Writing – original draft, Writing – review & editing. CL: Writing – original draft, Writing – review & editing.
