# Supplementary material for: Effect of reducing saturated fat intake on cardiovascular disease in adults: an umbrella review
Source: Front Public Health. 2024 Jun 3;12:1396576. doi: 10.3389/fpubh.2024.1396576 (PMC11180890; doi:10.3389/fpubh.2024.1396576)
Supplement: Supplementary file 1 [file Data_Sheet_1.doc]

**Supplementary material**

**Supplementary Table 1**. **Search strategy for each database.**

| **Base de datos: Medline (PubMed)** | | |
| --- | --- | --- |
| **Fecha de búsqueda:** 01 de diciembre de 2022 | | |
| **Nº** | **Término** | **Resultados** |
| 1 | Saturated Fatty Acid*[tiab] | 12,304 |
| 2 | dietary fats [mh] | 96,924 |
| 3 | ((fat [tiab] or fats [tiab] or fatty [tiab]) AND saturated [tiab]) OR SFA [ti] | 30,047 |
| 4 | intake [tiab] OR dietary [tiab] | 509,653 |
| 5 | #3 AND #4 | 12,862 |
| 6 | #1 OR #2 OR #5 | 110,164 |
| 7 | "cardiovascular disease" [tiab] OR "CVDs" [tiab] OR CVD [tiab] | 178,489 |
| 8 | "Cardiovascular Diseases" [mh] | 2,664,617 |
| 9 | "Heart Disease Risk Factors" [mh] | 5,095 |
| 10 | "cardiovascular health" [tiab] OR "cardiovascular outcomes" [tiab] OR "cardiovascular event*" [tiab] | 65,465 |
| 11 | "cardiometabolic disease*" [tiab] OR "cardiometabolic health" [tiab] | 5,545 |
| 12 | mortality [tiab] OR Mortality [mh] OR morbidity [tiab] OR Morbidity [mh] | 1,886,363 |
| 13 | "myocardial infarction" [tiab] OR "Myocardial Infarction" [mh] | 275,788 |
| 14 | stroke [tiab] OR Stroke [mh] | 336,557 |
| 15 | "coronary heart disease" [tiab] OR CHD [tiab] OR "Coronary Disease" [tiab] | 86,263 |
| 16 | cholesterol [tiab] OR Cholesterol [mh] | 316,640 |
| 17 | triglyceride* [tiab] OR Triglycerides [mh] | 164,078 |
| 18 | "lipid profile*" [tiab] OR "blood lipids" [tiab] | 45,140 |
| 19 | "blood pressure" [tiab] OR Blood Pressure [mh] OR systolic [tiab] OR diastolic [tiab] or Hypertension [mh] OR Hypertensi* [tiab] | 967,201 |
| 20 | Metabolic Syndrome [mh] OR metabolic [tiab] | 622,685 |
| 21 | Body Mass Index [mh] OR "body mass index" [tiab] OR BMI [tiab] OR "body weight" [mh] | 748,260 |
| 22 | overweight [tiab] OR obesity [tiab] OR overweight [mh] | 413,628 |
| 23 | Glycemic Index [mh] or Blood Glucose [mh] OR diabet* [tiab] OR glycem* [tiab] OR glycaem* [tiab] OR glucose [tiab] OR "Diabetes Mellitus" [mh] OR Glycemic Control [mh] | 1,201,676 |
| 24 | "Major adverse cardiovascular event*" [tiab] OR "Major adverse cardiac event*" [tiab] OR MACE [tiab] OR MACEs [tiab] | 17,359 |
| 25 | #7 OR #8 OR #9 OR #10 OR #11 OR #12 OR #13 OR #14 OR #15 OR #16 OR #17 OR #18 OR #19 OR #20 OR #21 OR #22 OR #23 OR #24 | 6,503,398 |
| 26 | "systematic review*" [tiab] OR "meta-analysis" [tiab] OR systematic [sb] OR meta-analysis [pt] | 413,289 |
| 27 | "2012/12/01 00:00":"2022/12/01 05:00" [dp] | 12,426,833 |
| 28 | #6 AND #25 AND #26 AND #27 AND medline [sb] | 706 |

| **Base de datos: Scopus** | | |
| --- | --- | --- |
| **Fecha de búsqueda:** 01 de diciembre de 2022 | | |
| **Nº** | **Término** | **Resultados** |
| 1 | TITLE-ABS ("saturated fatty acid*") OR TITLE-ABS (((((fat OR fats OR fatty) AND saturated) OR sfa) AND (intake OR dietary))) | 32,064 |
| 2 | TITLE-ABS ((cardi* AND (disease OR health OR outcomes OR event* OR effect*)) OR cvds OR cvd OR "myocardial infarction" OR stroke OR (coronary AND disease) OR mace OR maces OR "blood pressure" OR systolic OR diastolic OR hypertensi* OR cholesterol OR triglyceride* OR "lipid profile*" OR "blood lipids" OR mortality OR morbidity) | 3,722,408 |
| 3 | TITLE-ABS ("systematic review*" OR review* OR "meta-analysis") | 4,667,643 |
| 4 | (TITLE-ABS ("saturated fatty acid*") OR TITLE-ABS (((((fat OR fats OR fatty) AND saturated) OR sfa) AND (intake OR dietary)))) AND (TITLE-ABS ((cardi* AND (disease OR health OR outcomes OR event* OR effect*)) OR cvds OR cvd OR "myocardial infarction" OR stroke OR (coronary AND disease) OR mace OR maces OR "blood pressure" OR systolic OR diastolic OR hypertensi* OR cholesterol OR triglyceride* OR "lipid profile*" OR "blood lipids" OR mortality OR morbidity)) AND (TITLE-ABS ("systematic review*" OR review* OR "meta-analysis")) | 1,119 |
| 5 | (TITLE-ABS ("saturated fatty acid*") OR TITLE-ABS (((((fat OR fats OR fatty) AND saturated) OR sfa) AND (intake OR dietary)))) AND (TITLE-ABS ((cardi* AND (disease OR health OR outcomes OR event* OR effect*)) OR cvds OR cvd OR "myocardial infarction" OR stroke OR (coronary AND disease) OR mace OR maces OR "blood pressure" OR systolic OR diastolic OR hypertensi* OR cholesterol OR triglyceride* OR "lipid profile*" OR "blood lipids" OR mortality OR morbidity)) AND (TITLE-ABS ("systematic review*" OR review* OR "meta-analysis")) AND PUBYEAR > 2011 AND PUBYEAR < 2023 | 641 |

| **Base de datos: Cochrane Library** | | |
| --- | --- | --- |
| **Fecha de búsqueda:** 01 de diciembre de 2022 | | |
| **Nº** | **Término** | **Resultados** |
| #1 | ("Saturated Fatty Acid*):ti,ab,kw | 571 |
| #2 | MeSH descriptor: [Dietary Fats] explode all trees | 8067 |
| #3 | ((((fat or fats or fatty) and saturated) or SFA) and (intake or dietary)):ti,ab,kw | 2741 |
| #4 | #1 or #2 or #3 | 9878 |
| #5 | ("cardiovascular disease" or "CVDs" or "CVD"):ti,ab,kw | 26932 |
| #6 | MeSH descriptor: [Cardiovascular Diseases] explode all trees | 119381 |
| #7 | MeSH descriptor: [Heart Disease Risk Factors] explode all trees | 330 |
| #8 | ("cardiovascular health" or "cardiovascular outcomes" or "cardiovascular event*"):ti,ab,kw | 5791 |
| #9 | ("cardiometabolic disease*" or "cardiometabolic health"):ti,ab,kw | 963 |
| #10 | (mortality or morbidity):ti,ab,kw | 125823 |
| #11 | MeSH descriptor: [Mortality] explode all trees | 14101 |
| #12 | MeSH descriptor: [Morbidity] explode all trees | 15997 |
| #13 | ("myocardial infarction"):ti,ab,kw | 33595 |
| #14 | MeSH descriptor: [Myocardial Infarction] explode all trees | 11874 |
| #15 | (stroke):ti,ab,kw | 64616 |
| #16 | MeSH descriptor: [Stroke] explode all trees | 11922 |
| #17 | ("coronary heart disease" or CHD or "Coronary Disease"):ti,ab,kw | 16465 |
| #18 | (cholesterol):ti,ab,kw | 40574 |
| #19 | MeSH descriptor: [Cholesterol] explode all trees | 10701 |
| #20 | (triglyceride*):ti,ab,kw | 25099 |
| #21 | MeSH descriptor: [Triglycerides] explode all trees | 6686 |
| #22 | ("lipid profile*" or "blood lipids"):ti,ab,kw | 10896 |
| #23 | ("blood pressure" or systolic or diastolic or Hypertensi*):ti,ab,kw | 152531 |
| #24 | MeSH descriptor: [Blood Pressure] explode all trees | 28794 |
| #25 | MeSH descriptor: [Hypertension] explode all trees | 20148 |
| #26 | MeSH descriptor: [Metabolic Syndrome] explode all trees | 2131 |
| #27 | (metabolic):ti,ab,kw | 48621 |
| #28 | MeSH descriptor: [Body Mass Index] explode all trees | 11017 |
| #29 | MeSH descriptor: [Body Weight] explode all trees | 31782 |
| #30 | ("body mass index" or BMI):ti,ab,kw | 76876 |
| #31 | (overweight or obesity):ti,ab,kw | 49358 |
| #32 | MeSH descriptor: [Overweight] explode all trees | 19364 |
| #33 | MeSH descriptor: [Glycemic Index] explode all trees | 843 |
| #34 | MeSH descriptor: [Blood Glucose] explode all trees | 17663 |
| #35 | MeSH descriptor: [Diabetes Mellitus] explode all trees | 35999 |
| #36 | MeSH descriptor: [Glycemic Control] explode all trees | 261 |
| #37 | (diabet* or glycem* or glycaem* or glucose):ti,ab,kw | 145336 |
| #38 | ("Major adverse cardiovascular event*" or "Major adverse cardiac event*" or MACE or MACEs):ti,ab,kw | 4708 |
| #39 | #5 OR #6 OR #7 OR #8 OR #9 OR #10 OR #11 OR #12 OR #13 OR #14 OR #15 OR #16 OR #17 OR #18 OR #19 OR #20 OR #21 OR #22 OR #23 OR #24 OR #25 OR #26 OR #27 OR #28 OR #29 OR #30 OR #31 OR #32 OR #33 OR #34 OR #35 OR #36 OR #37 OR #38 | 568338 |
| #40 | #5 and #39 with Cochrane Library publication date from Dec 2012 to Dec 2022, in Cochrane Reviews | 36 |

| **Base de datos: EMBASE** | | |
| --- | --- | --- |
| **Fecha de búsqueda:** 01 de diciembre de 2022 | | |
| **Nº** | **Término** | **Resultados** |
| #1 | 'saturated fatty acid*':ab,ti | 14366 |
| #2 | 'dietary fats'/exp | 57086 |
| #3 | ((fat:ab,ti OR fats:ab,ti OR fatty:ab,ti) AND saturated:ab,ti OR sfa:ab,ti) AND (intake:ab,ti OR dietary:ab,ti) | 16888 |
| #4 | #1 OR #2 OR #3 | 75253 |
| #5 | 'cardiovascular disease':ab,ti OR 'cvds':ab,ti OR 'cvd':ab,ti | 246832 |
| #6 | 'cardiovascular disease'/exp | 5146706 |
| #7 | 'heart disease risk factor'/exp | 220 |
| #8 | 'cardiovascular health':ab,ti OR 'cardiovascular outcomes':ab,ti OR 'cardiovascular event*':ab,ti | 103885 |
| #9 | 'cardiometabolic disease*':ab,ti OR 'cardiometabolic health':ab,ti | 6980 |
| #10 | mortality:ab,ti OR morbidity:ab,ti | 1621946 |
| #11 | 'mortality'/exp OR 'morbidity'/exp | 1518560 |
| #12 | 'myocardial infarction':ab,ti OR 'heart infarction'/exp | 490634 |
| #13 | 'cerebrovascular accident'/exp OR stroke:ab,ti | 579360 |
| #14 | 'coronary heart disease':ab,ti OR chd:ab,ti OR 'coronary disease':ab,ti | 115126 |
| #15 | 'cholesterol'/exp OR cholesterol:ab,ti | 486260 |
| #16 | 'triacylglycerol'/exp OR triglyceride*:ab,ti | 289709 |
| #17 | 'lipid profile*':ab,ti OR 'blood lipids':ab,ti | 65624 |
| #18 | 'blood pressure':ab,ti OR systolic:ab,ti OR diastolic:ab,ti OR hypertensi*:ab,ti | 1216819 |
| #19 | 'blood pressure'/exp OR 'hypertension'/exp | 1381759 |
| #20 | 'metabolic syndrome x'/exp OR metabolic:ab,ti | 825121 |
| #21 | 'body mass'/exp OR 'body weight'/exp OR 'obesity'/exp OR 'body mass index':ab,ti OR bmi:ab,ti OR overweight:ab,ti OR obesity:ab,ti | 1766879 |
| #22 | 'glycemic index'/exp OR 'glucose blood level'/exp OR 'diabetes mellitus'/exp OR 'glycemic control'/exp OR diabet*:ab,ti OR glycem*:ab,ti OR glycaem*:ab,ti OR glucose:ab,ti | 1879941 |
| #23 | 'major adverse cardiovascular event*':ab,ti OR 'major adverse cardiac event*':ab,ti OR mace:ab,ti OR maces:ab,ti | 34374 |
| #24 | #5 OR #6 OR #7 OR #8 OR #9 OR #10 OR #11 OR #12 OR #13 OR #14 OR #15 OR #16 OR #17 OR #18 OR #19 OR #20 OR #21 OR #22 OR #23 | 9547029 |
| #25 | 'systematic review'/exp OR 'systematic review (topic)'/exp OR 'meta analysis'/exp OR 'meta analysis (topic)'/exp OR 'systematic review':ab,ti OR 'meta analysis':ab,ti | 641071 |
| #26 | #4 AND #24 AND #25 | 1318 |
| #27 | #4 AND #24 AND #25 AND [01-12-2012]/sd NOT [01-12-2022]/sd | 996 |

| **Base de datos: LILACS** | | |
| --- | --- | --- |
| **Fecha de búsqueda:** 01 de diciembre de 2022 | | |
| **Nº** | **Término** | **Resultados** |
| #1 | "Saturated Fatty Acid$" OR [MH] "Dietary Fats" OR ((((fat OR fats OR fatty) AND saturated) OR SFA) AND (intake OR dietary)) [Palabras] | 761 |
| #2 | "systematic review" OR review OR "meta-analysis" [Palabras] | 62109 |
| #3 | #1 AND #2 | 48 |

**Supplementary Table 2**. **List of excluded studies with reasons.**

| **No.** | **Author, year** | **Reason for exclusion** | **Reference** |
| --- | --- | --- | --- |
| 1 | Schwingshackl, 2013 | Wrong population | Schwingshackl L, Hoffmann G. Comparison of effects of long-term low-fat vs high-fat diets on blood lipid levels in overweight or obese patients: a systematic review and meta-analysis. J Acad Nutr Diet. 2013 Dec;113(12):1640-61. |
| 2 | O'Sullivan, 2013 | Wrong population | O'Sullivan TA, Hafekost K, Mitrou F, Lawrence D. Food sources of saturated fat and the association with mortality: a meta-analysis. Am J Public Health. 2013 Sep;103(9):e31-42. |
| 3 | Sadeghi, 2019 | Wrong study design | Sadeghi A, Shab-Bidar S, Parohan M, Djafarian K. Dietary Fat Intake and Risk of Ovarian Cancer: A Systematic Review and Dose-Response Meta-Analysis of Observational Studies. Nutr Cancer. 2019;71(6):939-953. |
| 4 | Clifton,2017 | Wrong study design | Clifton PM, Keogh JB. A systematic review of the effect of dietary saturated and polyunsaturated fat on heart disease. Nutr Metab Cardiovasc Dis. 2017 Dec;27(12):1060-1080. |
| 5 | Hamley,2017 | Wrong study design | Hamley S. The effect of replacing saturated fat with mostly n-6 polyunsaturated fat on coronary heart disease: a meta-analysis of randomised controlled trials. Nutr J. 2017 May 19;16(1):30. |
| 6 | Yao,2015 | Wrong study design | Yao X, Tian Z. Saturated, Monounsaturated and Polyunsaturated Fatty Acids Intake and Risk of Pancreatic Cancer: Evidence from Observational Studies. PLoS One. 2015 Jun 25;10(6): e0130870. |
| 7 | Schwingshackl,2022 | Wrong study design | Schwingshackl L, Heseker H, Kiesswetter E, Koletzko B. Dietary fat and fatty foods in the prevention of non-communicable diseases: A review of the evidence. Trends in Food Science & Technology. 2022;128 (0):173-184. |
| 8 | Szczerba,2021 | Wrong publication type | Szczerba E, Neuenschwander M, Schiemann T, Schlesinger S. Biomarkers of saturated fatty acid and incidence of type 2 diabetes: a systematic review and dose-response meta-analysis of prospective observational studies. Diabetologie und Stoffwechsel. 2021;16(S 01): S44. |
| 9 | Fretts,2017 | Wrong publication type | Fretts A, Imamura F, Yu C, Frazier-Wood AC, Lankinen M, Rajaobelina K, et al. Abstract MP014: Very Long Chain Saturated Fatty Acids and Diabetes Risk: Meta-Analysis of Cohort Studies in the FORCE Consortium. Circulation, 2017;135 (1): AMP014. |
| 10 | Hooper,2016 | Wrong publication type | Hooper L, Summerbell CD, Thompson R, Sills D, Roberts FG, Moore HJ, Smith GD. Reduced or modified dietary fat for preventing cardiovascular disease. Sao Paulo Medical Journal. 2016;134(2):182-3. |
| 11 | Sun.2014 | Wrong publication type | Sun Y, Neelakantan N, Wu Y, Van Dam RM. Effects of palm oil consumption on blood lipids: A meta-analysis of clinical trials. In Circulation Conference: American Heart Association’s Epidemiology and Prevention/Nutrition, Physical Activity, and Metabolism.2014;129. |
| 12 | Imamura,2013 | Wrong publication type | Imamura F, Micha R, Wu JH, de Oliveira Otto MC, Otite FO, Abioye AI, Mozaffarian D. Abstract 003:Effects of saturated, polyunsaturated, and monounsaturated fat on blood glucose, insulin sensitivity, and 2 cell function: A systematic review and meta-analysis of 84 randomized controlled feeding trials. Circulation, 2013;127 (12): AMP003. |
| 13 | Thomas,2013 | Wrong publication type | Thomas P, Mushtaq S. Saturated fatty acid intake as a risk factor for cardiovascular disease in affluent, healthy Caucasian adults: a systematic review and meta-analysis. Proceedings of the Nutrition Society,2013. 72(OCE4): E238. |
| 14 | Schwingshackl,2012 | Wrong publication type | Schwingshackl L, Hoffmann G. Comparison of long-term low-fat versus high-fat diets on blood lipids: a systematic review and meta-analysis. Proceedings of the Nutrition Society,2012. 71(OCE3):E220. |
| 15 | Nguyen,2021 | Wrong publication type | Nguyen S, Li H, Yu D, Cai H, Gao J, Gao Y et al. Dietary fatty acids and colorectal cancer risk in men: A report from the Shanghai Men's Health Study and a meta‐analysis. International Journal of Cancer.2021; 148(1):77-89. |

**Supplementary Table 3**. **List of excluded outcomes due to overlap.**

| **No.** | **Author, year** | **Outcomes** | **Reference** |
| --- | --- | --- | --- |
| 1 | Kim, 2021 | All-cause mortality | Kim Y, Je Y, Giovannucci EL. Association between dietary fat intake and mortality from all-causes, cardiovascular disease, and cancer: A systematic review and meta-analysis of prospective cohort studies. Clin Nutr. 2021 Mar;40(3):1060-1070. |
| 2 | de Souza, 2015 | All-cause mortality | de Souza RJ, Mente A, Maroleanu A, Cozma AI, Ha V, Kishibe T, Uleryk E, Budylowski P, Schünemann H, Beyene J, Anand SS. Intake of saturated and trans unsaturated fatty acids and risk of all cause mortality, cardiovascular disease, and type 2 diabetes: systematic review and meta-analysis of observational studies. BMJ. 2015 Aug 11;351:h3978. |
| 3 | de Souza, 2015 | Cardiovascular disease mortality | de Souza RJ, Mente A, Maroleanu A, Cozma AI, Ha V, Kishibe T, Uleryk E, Budylowski P, Schünemann H, Beyene J, Anand SS. Intake of saturated and trans unsaturated fatty acids and risk of all cause mortality, cardiovascular disease, and type 2 diabetes: systematic review and meta-analysis of observational studies. BMJ. 2015 Aug 11;351:h3978. |
| 4 | Mazidi, 2020 | Cardiovascular disease mortality | Mazidi M, Mikhailidis DP, Sattar N, Toth PP, Judd S, Blaha MJ, Hernandez AV, Penson PE, Banach M; International Lipid Expert Panel (ILEP) & Lipid and Blood Pressure Meta-analysis Collaboration (LBPMC) Group. Association of types of dietary fats and all-cause and cause-specific mortality: A prospective cohort study and meta-analysis of prospective studies with 1,164,029 participants. Clin Nutr. 2020 Dec;39(12):3677-3686. |
| 5 | de Souza, 2015 | Coronary heart disease mortality | de Souza RJ, Mente A, Maroleanu A, Cozma AI, Ha V, Kishibe T, Uleryk E, Budylowski P, Schünemann H, Beyene J, Anand SS. Intake of saturated and trans unsaturated fatty acids and risk of all cause mortality, cardiovascular disease, and type 2 diabetes: systematic review and meta-analysis of observational studies. BMJ. 2015 Aug 11;351:h3978. |
| 6 | Harcombe, 2016 | Coronary heart disease mortality | Harcombe Z, Baker JS, Davies B. Evidence from prospective cohort studies does not support current dietary fat guidelines: a systematic review and meta-analysis British Journal of Sports Medicine 2017;51:1743-1749. |
| 7 | Mazidi, 2020 | Stroke mortality | Mazidi M, Mikhailidis DP, Sattar N, Toth PP, Judd S, Blaha MJ, Hernandez AV, Penson PE, Banach M; International Lipid Expert Panel (ILEP) & Lipid and Blood Pressure Meta-analysis Collaboration (LBPMC) Group. Association of types of dietary fats and all-cause and cause-specific mortality: A prospective cohort study and meta-analysis of prospective studies with 1,164,029 participants. Clin Nutr. 2020 Dec;39(12):3677-3686. |
| 8 | Kang, 2020 | Stroke | Kang ZQ, Yang Y, Xiao B. Dietary saturated fat intake and risk of stroke: Systematic review and dose-response meta-analysis of prospective cohort studies. Nutr Metab Cardiovasc Dis. 2020 Feb 10;30(2):179-189. |
| 9 | Cheng, 2016 | Ischemic stroke | Cheng P, Wang J, Shao W, Liu M, Zhang H. Can dietary saturated fat be beneficial in prevention of stroke risk? A meta-analysis. Neurol Sci. 2016 Jul;37(7):1089-98. |
| 10 | Kang, 2020 | Ischemic stroke | Kang ZQ, Yang Y, Xiao B. Dietary saturated fat intake and risk of stroke: Systematic review and dose-response meta-analysis of prospective cohort studies. Nutr Metab Cardiovasc Dis. 2020 Feb 10;30(2):179-189. |
| 11 | Muto, 2018 | Ischemic stroke | Muto M, Ezaki O. High Dietary Saturated Fat is Associated with a Low Risk of Intracerebral Hemorrhage and Ischemic Stroke in Japanese but not in Non-Japanese: A Review and Meta-Analysis of Prospective Cohort Studies. J Atheroscler Thromb. 2018 May 1;25(5):375-392. |
| 12 | Cheng, 2016 | Hemorrhagic stroke | Cheng P, Wang J, Shao W, Liu M, Zhang H. Can dietary saturated fat be beneficial in prevention of stroke risk? A meta-analysis. Neurol Sci. 2016 Jul;37(7):1089-98. |
| 13 | Kang, 2020 | Intracranial hemorrhage | Kang ZQ, Yang Y, Xiao B. Dietary saturated fat intake and risk of stroke: Systematic review and dose-response meta-analysis of prospective cohort studies. Nutr Metab Cardiovasc Dis. 2020 Feb 10;30(2):179-189. |
| 14 | Qiu, 2016 | Ovarian Cancer | Qiu W, Lu H, Qi Y, Wang X. Dietary fat intake and ovarian cancer risk: a meta-analysis of epidemiological studies. Oncotarget. 2016 Jun 14;7(24):37390-37406. |
| 15 | Neuenschwander, 2020 | Type 2 diabetes | Neuenschwander M, Barbaresko J, Pischke CR, Iser N, Beckhaus J, Schwingshackl L, Schlesinger S. Intake of dietary fats and fatty acids and the incidence of type 2 diabetes: A systematic review and dose-response meta-analysis of prospective observational studies. PLoS Med. 2020 Dec 2;17(12): e1003347. |
| 16 | de Souza, 2015 | Type 2 diabetes | de Souza RJ, Mente A, Maroleanu A, Cozma AI, Ha V, Kishibe T, Uleryk E, Budylowski P, Schünemann H, Beyene J, Anand SS. Intake of saturated and trans unsaturated fatty acids and risk of all cause mortality, cardiovascular disease, and type 2 diabetes: systematic review and meta-analysis of observational studies. BMJ. 2015 Aug 11;351:h3978. |

**Supplementary Table 4. Characteristics of meta-analyses of cohorts studies studying saturated fat intake.**

| **Author (year)** | **Population** | **Type of exposure** | **Comparator** | **Study follow-up range (years)** | **No. of included studies** | **Total participants** | **Outcomes** ^a^ | **AMSTAR-2 rating** |
| --- | --- | --- | --- | --- | --- | --- | --- | --- |
| Kim (2021) ^b^ | Adults(>20 years) without pre-existing disease at baseline | Higher SFA intake: Higher intake category  g/day (range: 13.7 to 34.7)  % total energy (range: 2.5% to 8.7%) | Reduced SFA intake:  Lowest intake category  g/day (range: 24.3 to 67.5)  % total energy (range: 7.3% to 17.9%) | 6 to 32 | 15 | 926,897 | 1,2,3 | Critically low |
| de Souza (2015) ^b,c,d,k^ | Adults (>16 years) | Higher SFA intake: Higher intake category  g/day (range: 7 to 55.7)  % total energy (range: 0.7% to 36.1%) | Reduced SFA intake:  Lowest intake category  g/day (range: 15.4 to 86.6)  % total energy (range: 1.5% to 44.8%) | 1 to 32 | 41 | 173,444 | 1,2,4,5,6,7 | Low |
| Mazidi (2020) ^c,e^ | Adults (>18 years) | Higher SFA intake: According to percentiles, gr/day, % energy, increase in different units | Reduced SFA intake: According to percentiles, gr/day, % energy, increase in different units | 3.7 to 32 | 29 | 1,164,029 | 1,2,4,8 | Critically low |
| Harcombe (2016) ^d^ | Adults (30-79 years) | Higher SFA intake: Higher intake category  g/day (34.7)  % total energy (range: 2.4% to 16.3%) | Reduced SFA intake:  Lowest intake category  g/day (67.5)  % total energy (range: 8.2% to 29.6%) | 6 to 20 | 7 | 89,801 | 4 | Critically low |
| Cheng (2016) ^g,h^ | Adults (20-89 years) | Higher SFA intake: Higher intake category (range: 15.4 to 36 gr/day) | Dieta baja en SFA : Lowest intake category (range: 7 to 20 gr/day) | 7.6 to 23 | 15 | 476,569 | 6,8,9,10 | Critically low |
| Brennan (2017) | Adults (19-75 years) | Higher SFA intake: Higher intake category | Reduced SFA intake: Lowest intake category | 5.5 to 18 | 4 | 3675 | 11 | Critically low |
| Zhu (2019) | Adults (>18 years) | Higher SFA intake: Higher intake category | Reduced SFA intake: Lowest intake category | NR | 56 | NR | 12 | Critically low |
| Kang (2020) ^f,g,i^ | Adults (>18 years)  (excluded participants who had suffered a stroke event before recruitment) | Higher SFA intake: Higher intake category (range: 15.4 to 50.4 gr/day) | Reduced SFA intake: Lowest intake category (range: 5.2 to 26.8 gr/day) | 7.4 to 20 | 14 | 462,268 | 6,10,13,14 | Low |
| Muto (2018) ^g^ | Adults (34-89 years) | Higher SFA intake: Higher intake category | Reduced SFA intake: Lowest intake category | 7.6 to 20 | 11 | 415,731 | 6,9 | Critically low |
| Kim (2018) | Adults (34-69 years) | Higher SFA intake: Higher intake category (range: 11.9 to 86.6 gr/day) | Reduced SFA intake: Lowest intake category (range: 5.9 to 35.6 gr/day) | 3.3 to 32 | 9 | 353,531 | 15 | Critically low |
| Qiu (2016) ^j^ | Adults (>18 years) | Higher SFA intake: Higher intake category | Reduced SFA intake: Lowest intake category | 8 to 19 | 6 | 745,748 | 17 | Critically low |
| Khodavandi (2021) | Adults (19-93 years) | Higher SFA intake: Higher intake category (range: 17.4 to 21.6 gr/day) | Reduced SFA intake: Lowest intake category (range: 23 to 35 gr/day) | 8 to 28 | 8 | 1,330,834 | 17 | Critically low |
| Zhao (2021) | Adults >50 years | Higher SFA intake: Higher intake category | Reduced SFA intake: Lowest intake category | 11.4 to 26.6 | 5 | 1,180,214 | 18 | Critically low |
| Lodi (2022) | Adults (>18 years) | Higher SFA intake: Higher intake category | Reduced SFA intake: Lowest intake category | 5 to 16 | 8 | 595,253 | 19 | Critically low |
| Zhao (2016) | Adults (>18 years) | Higher SFA intake: Higher intake category (range: 29.3 to 102.8 gr/day) | Reduced SFA intake: Lowest intake category (range: 17.3 to 85.9 gr/day) | 13 to 26 | 3 | 528,343 | 20 | Critically low |
| Neuenschwander (2020) ^k^ | Adults (30-80 years) | Higher SFA intake: Higher intake category (range: 25.8 a 40.01 gr/day) | Reduced SFA intake: Lowest intake category (range: 12.37 to 33.3 gr/day) | 4.1 to 18 | 11 | 355,961 | 7 | Low |
| Gaeini (2022) | Adults (>18 years) | Higher SFA intake: Higher intake category | Reduced SFA intake: Lowest intake category | 4 to 17 | 13 | 361,686 | 7 | Critically low |
| Chowdhury (2014) | Adults (>18 years) from general populations or with estable cardiovascular disease | Higher SFA intake: Top third of baseline intake | Higher SFA intake: Bottom third of baseline intake | 1.3 to 30.7 | 20 | 283,963 | 5 | Critically low |

**Abbreviations: No:** Number; **AMSTAR-2:** A Measurement Tool to Assess Systematic Reviews; **SFA**: Saturated fatty acids; **gr**: Gramos.

1. 1 = All-causes mortality, 2= Cardiovascular mortality, 3= Cancer mortality, 4=Coronary heart disease mortality, 5= Coronary heart disease, 6=Ischemic stroke, 7= Type 2 diabetes, 8= Fatal stroke, 9= Hemorrhagic stroke, 10= Stroke events, 11= Breast cancer mortality, 12= Cardiovascular disease, 13= Subarachnoid hemorrhage, 14= Intracranial hemorrhage, 15= Colorectal cancer, 16= Pancreatic cancer, 17= Ovarian cancer, 18= Liver cancer, 19= Breast cancer, 20= Endometrial cancer.
2. Excluded by overlap to evaluate: All-cause mortality.
3. Excluded by overlap to evaluate: Cardiovascular disease mortality.
4. Excluded by overlap to evaluate: Coronary heart disease mortality.
5. Excluded by overlap to evaluate: Stroke mortality.
6. Excluded by overlap to evaluate: Stroke.
7. Excluded by overlap to evaluate: Ischemic stroke.
8. Excluded by overlap to evaluate: Hemorrhagic stroke.
9. Excluded by overlap to evaluate: Intracranial hemorrhage.
10. Excluded by overlap to evaluate: Ovarian Cancer.
11. Excluded by overlap to evaluate: Type 2 diabetes.

**Supplementary Table 5. Quality assessment ^a^.**

| **Author (year)** | **1. Did the research questions and inclusion criteria for the review include the components of**  **PICO?** | **2. Did the report of the review contain an explicit statement that the review methods were**  **established prior to the conduct of the review and did the report justify any significant**  **deviations from the protocol? ^b^** | **3. Did the review authors explain their selection of the study designs for inclusion in the review?** | **4. Did the review authors use a comprehensive literature search strategy? ^b^** | **5. Did the review authors perform study selection in duplicate?** | **6. Did the review authors perform data extraction in duplicate?** | **7. Did the review authors provide a list of excluded studies and justify the exclusions? ^b^** | **8. Did the review authors describe the included studies in adequate detail?** | **9. Did the review authors use a satisfactory technique for assessing the risk of bias in**  **individual studies that were included in the review? ^b^** | **10. Did the review authors report on the sources of funding for the studies included in the review?** | **11. If meta-analysis was performed did the review authors use appropriate methods for statistical**  **combination of results?^b^** | **12. If meta-analysis was performed, did the review authors assess the potential impact of risk of bias in**  **individual studies on the results of the meta-analysis or other evidence synthesis?** | **13. Did the review authors account for risk of bias in individual studies when interpreting/discussing**  **the results of the review? ^b^** | **14. Did the review authors provide a satisfactory explanation for, and discussion of, any**  **heterogeneity observed in the results of the review?** | **15. If they performed quantitative synthesis did the review authors carry out an adequate**  **investigation of publication bias (small study bias) and discuss its likely impact on the results**  **of the review? ^b^** | **16. Did the review authors report any potential sources of conflict of interest, including any**  **funding they received for conducting the review?** | **AMSTAR-2 score** | **AMSTAR-2 rating** |
| --- | --- | --- | --- | --- | --- | --- | --- | --- | --- | --- | --- | --- | --- | --- | --- | --- | --- | --- |
| Hooper (2020) | Yes | Yes | Yes | Yes | Yes | Yes | Yes | Yes | Yes | Yes | Yes | Yes | Yes | Yes | Yes | Yes | 16/16 | High |
| Imamura (2016) | Yes | Yes | Yes | Yes | Yes | Yes | No | Yes | Yes | Yes | Yes | No | No | Yes | Yes | Yes | 13/16 | Critically low |
| Hannon (2017) | Yes | Yes | Yes | No | No | No | Yes | Yes | Yes | Yes | No | No | No | Yes | Yes | Yes | 10/16 | Critically low |
| Kim (2021) | Yes | No | Yes | No | No | Yes | No | Yes | Yes | Yes | Yes | No | No | Yes | Yes | Yes | 10/16 | Critically low |
| de Souza (2015) | Yes | No | Yes | Yes | Yes | Yes | No | Yes | Yes | Yes | Yes | Yes | Yes | Yes | Yes | Yes | 15/16 | Low |
| Mazidi (2020) | Yes | No | Yes | Yes | Yes | Yes | No | Yes | Yes | Yes | Yes | No | No | No | Yes | Yes | 11/16 | Critically low |
| Harcombe (2016) | Yes | No | Yes | No | No | No | No | Yes | Yes | Yes | Yes | No | No | No | Yes | Yes | 8/16 | Critically low |
| Cheng (2016) | Yes | No | Yes | No | No | Yes | No | Yes | Yes | Yes | Yes | Yes | Yes | Yes | Yes | Yes | 12/16 | Critically low |
| Brennan (2017) | Yes | No | Yes | No | Yes | No | No | Yes | No | Yes | Yes | No | No | No | Yes | No | 7/16 | Critically low |
| Zhu (2019) | Yes | No | Yes | No | Yes | Yes | No | Yes | No | Yes | Yes | No | No | No | Yes | Yes | 9/16 | Critically low |
| Kang (2020) | Yes | Yes | Yes | Yes | Yes | Yes | No | Yes | Yes | Yes | Yes | Yes | Yes | Yes | Yes | Yes | 15/16 | Low |
| Muto (2018) | Yes | No | Yes | No | No | No | No | Yes | Yes | No | Yes | No | No | Yes | Yes | Yes | 8/16 | Critically low |
| Kim (2018) | Yes | No | Yes | No | Yes | Yes | No | Yes | Yes | Yes | Yes | No | No | Yes | Yes | Yes | 11/16 | Critically low |
| Qiu (2016) | Yes | No | Yes | No | No | No | No | Yes | No | Yes | Yes | No | No | No | Yes | Yes | 7/16 | Critically low |
| Khodavandi (2021) | Yes | No | Yes | Yes | No | Yes | Yes | Yes | No | Yes | Yes | No | No | No | No | Yes | 9/16 | Critically low |
| Zhao (2021) | Yes | No | Yes | Yes | Yes | Yes | Yes | Yes | Yes | Yes | Yes | No | No | No | Yes | Yes | 12/16 | Critically low |
| Lodi (2022) | Yes | No | Yes | No | No | Yes | No | Yes | No | Yes | Yes | No | No | No | No | Yes | 7/16 | Critically low |
| Zhao (2016) | Yes | No | Yes | No | Yes | Yes | No | Yes | No | No | Yes | No | No | No | Yes | No | 7/16 | Critically low |
| Neuenschwander (2020) | Yes | Yes | Yes | No | Yes | Yes | Yes | Yes | Yes | Yes | Yes | Yes | Yes | Yes | Yes | Yes | 15/16 | Low |
| Gaeini (2022) | Yes | No | Yes | No | Yes | Yes | No | Yes | Yes | Yes | Yes | Yes | Yes | Yes | Yes | Yes | 13/16 | Critically low |
| Chowdhury (2014) | Yes | Yes | Yes | Yes | No | Yes | No | Yes | Yes | Yes | Yes | No | No | No | Yes | Yes | 11/16 | Critically low |

**Abbreviations: AMSTAR-2:** A Measurement Tool to Assess Systematic Reviews.

1. Quality assessment using AMSTAR-2.
2. AMSTAR-2 critical domains.

**Supplementary Table 6. Summary of secondary findings of meta-analyses of randomized clinical trials studying saturated fat intake.**

| **Author (year)** | **Outcomes** | **Population** | **Type of intervention** | **Comparator** | **Study follow-up range** | **No. of included studies** | **Intervention****/comparator** | **Measures of effect** | **Effect size**  **(95%CI)** | **I^2^,%** | **AMSTA-2 rating** | **GRADE rating** |
| --- | --- | --- | --- | --- | --- | --- | --- | --- | --- | --- | --- | --- |
| Cancer | | | | | | | | | | | | |
| Hooper, 2020 | Cancer diagnoses | Adults  (≥18 years) ^a^ | Reduced SFA intake ^b^ | Higher SFA intake ^c^ | 3.7 to 8.1 years ^d^ | 4 | 2143 en 21028/3333 en 31266 | RR | 0.94   (0.83, 1.07) | 33 | High | NR |
| Glucose-insulin homeostasis | | | | | | | | | | | | |
| Hooper, 2020 | GTT, mmol/L | Adults (≥18 years) ^a^ | Reduced SFA intake ^b^ | Higher SFA intake ^c^ | 1.9 to 3 years ^d^ | 3 | 125/124 | MD | -1.69 (-2.55, -0.82) | 45 | High | NR |
| Hooper, 2020 | HOMA-IR | Adults (≥18 years) ^a^ | Reduced SFA intake ^b^ | Higher SFA intake ^c^ | Mean: 8.6 years | 1 | 1133/1699 | MD | 0.0 (-0.04, 0.04) | NA | High | NR |
| Imamura, 2016 | Fasting glucose, mmol/L | Adults (≥18 years) ^e^ | Exchanging SFA with MUFA | Intake without exchanging SFA | Mean: 4 weeks | 99 | NR | MD | -0.02 (-0.04, 0.00) | NR | Critically low | NR |
| Imamura, 2016 | Fasting glucose, mmol/L | Adults (≥18 years) ^e^ | Exchanging SFA with PUFA | Intake without exchanging SFA | Mean: 4 weeks | 99 | NR | MD | -0.04 (-0.07, -0.01) | NR | Critically low | NR |
| Imamura, 2016 | GTT, mmol/L | Adults (≥18 years) ^e^ | Exchanging SFA with MUFA | Intake without exchanging SFA | Mean: 4 weeks | 11 | NR | MD | -0.10 (-0.91, 0.70) | NR | Critically low | NR |
| Imamura, 2016 | GTT, mmol/L | Adults (≥18 years) ^e^ | Exchanging SFA with PUFA | Intake without exchanging SFA | Mean: 4 weeks | 11 | NR | MD | 0.26 (-0.34, 0.85) | NR | Critically low | NR |
| Imamura, 2016 | Haemoglobin A1c,% | Adults (≥18 years) ^e^ | Exchanging SFA with MUFA | Intake without exchanging SFA | Mean: 4 weeks | 23 | NR | MD | -0.12 (-0.19, -0.05) | NR | Critically low | NR |
| Imamura, 2016 | Haemoglobin A1c,% | Adults (≥18 years) ^e^ | Exchanging SFA with PUFA | Intake without exchanging SFA | Mean: 4 weeks | 23 | NR | MD | -0.15 (-0.23, -0.06) | NR | Critically low | NR |
| Imamura, 2016 | Fasting insulin, pmol/L | Adults (≥18 years) ^e^ | Exchanging SFA with MUFA | Intake without exchanging SFA | Mean: 4 weeks | 90 | NR | MD | 1.2 (0.6, 1.8) | NR | Critically low | NR |
| Imamura, 2016 | Fasting insulin, pmol/L | Adults (≥18 years) ^e^ | Exchanging SFA with PUFA | Intake without exchanging SFA | Mean: 4 weeks | 90 | NR | MD | -0.5 (-2.0, 1.1) | NR | Critically low | NR |
| Imamura, 2016 | 2 h insulin, pmol/L ^g^ | Adults (≥18 years) ^e^ | Exchanging SFA with MUFA | Intake without exchanging SFA | Mean: 4 weeks | 11 | NR | MD | -22.2 (-49.1, 4.6) | NR | Critically low | NR |
| Imamura, 2016 | 2 h insulin, pmol/L ^g^ | Adults (≥18 years) ^e^ | Exchanging SFA with PUFA | Intake without exchanging SFA | Mean: 4 weeks | 11 | NR | MD | -26.8 (-72.5, 18.9) | NR | Critically low | NR |
| Imamura, 2016 | C-peptide, nmol/L | Adults (≥18 years) ^e^ | Exchanging SFA with MUFA | Intake without exchanging SFA | Mean: 4 weeks | 7 | NR | MD | -0.01 (-0.03, 0.01) | NR | Critically low | NR |
| Imamura, 2016 | C-peptide, nmol/L | Adults (≥18 years) ^e^ | Exchanging SFA with PUFA | Intake without exchanging SFA | Mean: 4 weeks | 7 | NR | MD | -0.07 (-0.14, -0.01) | NR | Critically low | NR |
| Imamura, 2016 | HOMA-IR, % change | Adults (≥18 years) ^e^ | Exchanging SFA with MUFA | Intake without exchanging SFA | Mean: 4 weeks | 30 | NR | MD | -3.1 (-5.8, -0.4) | NR | Critically low | NR |
| Imamura, 2016 | HOMA-IR, % change | Adults (≥18 years) ^e^ | Exchanging SFA with PUFA | Intake without exchanging SFA | Mean: 4 weeks | 30 | NR | MD | -4.1 (-6.4, -1.6) | NR | Critically low | NR |
| Imamura, 2016 | Insulin sensitivity index, 10−5/(pmol/L)/min ^h^ | Adults (≥18 years) ^e^ | Exchanging SFA with MUFA | Intake without exchanging SFA | Mean: 4 weeks | 13 | NR | MD | 0.08 (-0.01, 0.17) | NR | Critically low | NR |
| Imamura, 2016 | Insulin sensitivity index, 10−5/(pmol/L)/min ^h^ | Adults (≥18 years) ^e^ | Exchanging SFA with PUFA | Intake without exchanging SFA | Mean: 4 weeks | 13 | NR | MD | 0.24 (-0.13, 0.61) | NR | Critically low | NR |
| Imamura, 2016 | Acute insulin response, pmol/L/min ^I^ | Adults (≥18 years) ^e^ | Exchanging SFA with MUFA | Intake without exchanging SFA | Mean: 4 weeks | 10 | NR | MD | -0.01 (-0.08, 0.06) | NR | Critically low | NR |
| Imamura, 2016 | Acute insulin response, pmol/L/min ^I^ | Adults (≥18 years) ^e^ | Exchanging SFA with PUFA | Intake without exchanging SFA | Mean: 4 weeks | 10 | NR | MD | 0.51 (0.20, 0.82) | NR | Critically low | NR |
| Lipidic profile | | | | | | | | | | | | |
| Hooper, 2020 | Total cholesterol,mmol/L | Adults  (≥18 years) ^a^ | Reduced SFA intake ^b^ | Higher SFA intake ^c^ | 1.5 to 9.3 years ^d^ | 13 | 3269/3846 | MD | -0.24 (-0.36, -0.13) | 60 | High | NR |
| Hooper, 2020 | LDL-cholesterol,mmol/L | Adults  (≥18 years) ^a^ | Reduced SFA intake ^b^ | Higher SFA intake ^c^ | 1.7 to 8.6 years ^d^ | 5 | 1361/1930 | MD | -0.19 (-0.33, -0.05) | 37 | High | NR |
| Hooper, 2020 | HDL-cholesterol,mmol/L | Adults  (≥18 years) ^a^ | Reduced SFA intake ^b^ | Higher SFA intake ^c^ | 1.7 to 8.6 years ^d^ | 6 | 2285/2862 | MD | -0.01 (-0.02, 0.01) | 0 | High | NR |
| Hooper, 2020 | Triglycerides | Adults  (≥18 years) ^a^ | Reduced SFA intake ^b^ | Higher SFA intake ^c^ | 1.7 to 8.6 years ^d^ | 7 | 1630/2215 | MD | -0.08 (-0.21, 0.04) | 51 | High | NR |
| Hooper, 2020 | Total cholesterol/HDL ratio | Adults  (≥18 years) ^a^ | Reduced SFA intake ^b^ | Higher SFA intake ^c^ | 1.9 to 8.6 years ^d^ | 3 | 1210/1775 | MD | -0.10 (-0.33, 0.13) | 24 | High | NR |
| Hooper, 2020 | LDL/HDL | Adults  (≥18 years) ^a^ | Reduced SFA intake ^b^ | Higher SFA intake ^c^ | 3 | 1 | 26/24 | MD | -0.36 (-0.92, 0.20) | NA | High | NR |
| Hannon, 2017 | Total cholesterol | Healthy adults  (≥18 years) ^f^ | Replacement of SFA with MUFA or PUFA | Intake without replacement in SFA | 4 and 28 weeks | 8 | NR | MD | -10.68 (-21.90, 0.53) | 95 | Critically low | NR |
| Hannon, 2017 | LDL-cholesterol | Healthy adults  (≥18 years) ^f^ | Replacement of SFA with MUFA or PUFA | Intake without replacement in SFA | 4 and 28 weeks | 8 | NR | MD | -8.7 (-19.17, 1.77) | 96 | Critically low | NR |
| Hannon, 2017 | HDL-cholesterol | Healthy adults  (≥18 years) ^f^ | Replacement of SFA with MUFA or PUFA | Intake without replacement in SFA | 4 and 28 weeks | 8 | NR | MD | 1.15 (-4.57, 6.86) | 98 | Critically low | NR |
| Hannon, 2017 | Triglycerides | Healthy adults  (≥18 years) ^f^ | Replacement of SFA with MUFA or PUFA | Intake without replacement in SFA | 4 a 15 weeks | 7 | NR | MD | -9.07 (-23.55, 5.42) | 96 | Critically low | NR |
| Body weight | | | | | | | | | | | | |
| Hooper, 2020 | Weight, kg | Adults  (≥18 years) ^a^ | Reduced SFA intake ^b^ | Higher SFA intake ^c^ | 1.7 to 8.1 years ^d^ | 6 | 17018/26044 | MD | -1.77 (-3.54, -0.01) | 77 | High | NR |
| Hooper, 2020 | BMI, kg/m2 | Adults  (≥18 years) ^a^ | Reduced SFA intake ^b^ | Higher SFA intake ^c^ | 1.7 to 9.3 years ^d^ | 6 | 17344/26550 | MD | -0.42 (-0.72, -0.12) | 62 | High | NR |
| Blood Pressure | | | | | | | | | | | | |
| Hooper, 2020 | Systolic Blood Pressure, mmHg | Adults  (≥18 years) ^a^ | Reduced SFA intake ^b^ | Higher SFA intake ^c^ | 1.9 to 8.6 years ^d^ | 5 | 1619/2193 | MD | -0.19 (-1.36, 0.97) | 0 | High | NR |
| Hooper, 2020 | Diastolic Blood Pressure, mmHg | Adults  (≥18 years) ^a^ | Reduced SFA intake ^b^ | Higher SFA intake ^c^ | 1.9 to 8.6 years ^d^ | 5 | 1619/2193 | MD | -0.36 (-1.03, 0.32) | 0 | High | NR |
| Quality of Life | | | | | | | | | | | | |
| Hooper, 2020 | Quality of Life ^j^ | Adults  (≥18 years) ^a^ | Reduced SFA intake ^b^ | Higher SFA intake ^c^ | Mean: 8.6 years | 1 | 15788/24342 | MD | 0.04 (0.01, 0.07) | NA | High | NR |

**Abbreviations:No:** Number**; CI:**Confidence intervals**;I^2^:**Statistic assessment of heterogeneity**;%:** Percentage; **AMSTAR-2:** A Measurement Tool to Assess Systematic Reviews**; GRADE:**Grading of Recommendations, Assessment, Development, and Evaluations **SFA:** Saturated fatty acids**; RR:** Relative risk; **NR:** Not reported; **MD:** Mean difference**;GTT**: glucose tolerance test, glucose at 2 hours; **HOMA-IR**: Homeostasis model assessment of insulin resistance;**NA**: Not applicable;**MUFA**:Monounsaturated fat;**PUFA**: polyunsaturated fat;**LDL**:Low density lipoprotein;**HDL**:High-density lipoprotein;**BMI**:Body mass index.

1. Adults (18 years or older, no upper age limit) at any risk of cardiovascular disease, with or without existing cardiovascular disease, using or not using lipid-lower in medication. Participants could be of either gender, but we excluded those who were acutely ill, pregnant or lactating.
2. By suggesting appropriate nutrient based or food-based aims, or which provided a general dietary aim, such as improving heart health or reducing total fat. The intervention had to be dietary advice, supplementation of fats, oils or modified or low-fat foods, or a provided diet.
3. Which could be a diet high in saturated fat, or a usual diet (not modified in SFA).
4. Range of mean years in trial.
5. Non‐pregnant.
6. Healthy adults (≥18 years) with criteria for overweight and obesity without diagnosis of metabolic disease.
7. 2 h post-oral-challenge insulin.
8. It is useful for measuring insulin sensitivity, which is the inverse (IR).
9. Gold-standard measures of β-cell function.
10. Quality of life was assessed at baseline using the SF-36 tool.

**Supplementary Table 7. Summary of secondary findings of meta-analyses of cohorts studying saturated fat intake.**

| **Author (year)** | **Outcomes** | **Population** | **Type of exposure** | **Comparator** | **Study follow-up range (years)** | **No. of**  **included**  **studies** | **Intervention****/comparator** | **Measures of effect** | **Effect size**  **(95%CI)** | **I^2^,%** | **AMSTA-2 rating** | **GRADE rating** |
| --- | --- | --- | --- | --- | --- | --- | --- | --- | --- | --- | --- | --- |
| Incidence of cancer | | | | | | | | | | | | |
| Kim (2018) | Colorectal cancer | Adultos de 34-69 años | Higher SFA intake: Higher intake category (range: 11.9 to 86.6 gr/day) | Reduced SFA intake: Lowest intake category (range: 5.9 to 35.6 gr/day) | 3.3 to 32 | 9 | NR | RR | 0.97  (0.86, 1.10) | 0 | Critically low | NR |
| Khodavandi (2021) | Ovarian Cancer | Adultos 19-93 años | Higher SFA intake: Higher intake category (range: 17.4 to 21.6 gr/day) | Reduced SFA intake: Lowest intake category  (range: 23 to 35 gr/day) | 8 to 28 | 8 | NR | RR | 1.11  (0.86, 1.10) | 6.6 | Critically low | NR |
| Zhao (2021) | Liver cancer | Adultos >50 años | Higher SFA intake: Higher intake category | Reduced SFA intake: Lowest intake category | 11.4 to 26.6 | 5 ^a^ | NR | RR | 1.34  (1.06, 1.69) | 16.9 | Critically low | NR |
| Lodi (2022) | Breast cancer | Adultos >18 años | Higher SFA intake: Higher intake category | Reduced SFA intake: Lowest intake category | 5 to 16 | 8 | NR | RR | 0.94  (0.74, 1.18) | 92 | Critically low | NR |
| Zhao (2016) | Endometrial cancer | Adultos | Higher SFA intake: Higher intake category  (range: 29.3 to 102.8 gr/day) | Reduced SFA intake: Lowest intake category (range: 17.3 to 85.9 gr/day) | 13 to 26 | 3 | NR | RR | 0.91 (0.80, 1.03) | 27.07 | Critically low | NR |
| Diabetes | | | | | | | | | | | | |
| Gaeini (2022) | Type 2 diabetes | Adultos >18 años | Higher SFA intake: Higher intake category | Reduced SFA intake: Lowest intake category | 4 to 17 | 13 | NR | HR | 0.99 (0.91, 1.09) | 54.4 | Critically low | NR |

**Abbreviations: No:** Number**; CI:** Confidence intervals**; I^2^:** Statistic assessment of heterogeneity**; %:** Percentage; **AMSTAR-2:** A Measurement Tool to Assess Systematic Reviews**; GRADE:** Grading of Recommendations, Assessment, Development, and Evaluations**; SFA:** Saturated fatty acids; **gr:** Gramos; **NR:** No reported**; RR:** Relative risk; **HR:** Hazard ratio**.**

1. Adjustment for confounding variables: Age, sex, year of interview, dietary protein, physical activity, alcohol, BMI, cigarette smoking, diabetes, education, aspirin use, type 2 diabetes, total coffee intake, total energy intake, fruit intake, vegetable intake, marital status, race and/or ethnicity, total energy from non alcohol sources, energy from other macronutrients, subscapular-to-triceps skinfold ratio.

**Supplementary Table 8**. **The GRADE assessment in meta-analyses of RCTs.**

**Reference:** Hooper L, Martin N, Jimoh OF, Kirk C, Foster E, Abdelhamid AS. Reduction in saturated fat intake for cardiovascular disease. Cochrane Database Syst Rev. 2020;5: Cd011737. doi: 10.1002/14651858.CD011737.pub2

| **№ of studies** | **Risk of bias** | **Inconsistency** | **Indirectness** | **Imprecision** | **Others** | **Certainty** |  |
| --- | --- | --- | --- | --- | --- | --- | --- |
|  |  |  |  |  |  |  |  |
| **All-cause mortality (follow-up: range 1.5 years to 8.6 years)** | | | | | | |  |
| 12 | not serious | not serious | not serious | serious^a^ | none | ⨁⨁⨁◯ |  |
|  |  |  |  |  |  | Moderate |  |
| **Cardiovascular mortality (follow-up: range 1.5 years to 8.6 years)** | | | | | | |  |
| 11 | not serious | not serious | not serious | serious^b^ | none | ⨁⨁⨁◯ |  |
|  |  |  |  |  |  | Moderate |  |
| **Coronary heart disease mortality (follow-up: range 1.5 years to 8.6 years)** | | | | | | |  |
| 9 | not serious | not serious | not serious | very serious^c^ | none | ⨁⨁◯◯ |  |
|  |  |  |  |  |  | Low |  |
| **Combined cardiovascular events (follow-up: range 1.5 years to 8.6 years)** | | | | | | |  |
| 12 | not serious | not serious | not serious | not serious | publication bias strongly suspected^d^ | ⨁⨁⨁◯ |  |
|  |  |  |  |  |  | Moderate |  |
| **Myocardial infarction (follow-up: range 1.5 years to 8.6 years)** | | | | | | |  |
| 11 | serious^e^ | not serious | not serious | serious^a^ | publication bias strongly suspected^f^ | ⨁◯◯◯ |  |
|  |  |  |  |  |  | Very low |  |
| **Non-fatal myocardial infarction (follow-up: range 1.5 years to 8.6 years)** | | | | | | |  |
| 8 | serious^g^ | not serious | not serious | serious^a^ | none | ⨁⨁◯◯ |  |
|  |  |  |  |  |  | Low |  |
| **Coronary heart disease events (follow-up: range 1.9 years to 8.6 years)** | | | | | | |  |
| 11 | serious^e^ | serious^h^ | not serious | serious^a^ | none | ⨁◯◯◯ |  |
|  |  |  |  |  |  | Very low |  |
| **Stroke (follow-up: range 1.5 years to 8.6 years)** | | | | | | |  |
| 7 | serious^g^ | not serious | not serious | very serious^c^ | none | ⨁◯◯◯ |  |
|  |  |  |  |  |  | Very low |  |

**Explanations**

^a^ The 95% CI includes both no effect and a benefit.

^b^ The 95% CI includes both harm and benefit.

^c^ The 95% CI includes both important benefits and important harms.

^d^ Publication bias. The funnel plot, and comparison of fixed- and random-effects meta-analyses suggested some small-study (publication) bias.

^e^ Limiting trials to those at low summary risk of bias moved the RR slightly towards 1.0, suggesting little or no effect on total MI.

^f^ The funnel plot, and comparison of fixed- and random-effects meta-analyses suggested some small-study (publication) bias.

^g^ Limiting trials to those at low summary risk of bias moved the RR slightly away from 1.0, suggesting that reducing SFA reduces the risk of non-fatal MI. This was also seen in several other sensitivity analyses.

^h^ Heterogeneity was high, *I^2^* = 65%.

**Supplementary Table 9**. **The GRADE assessment in meta-analyses of cohort studies.**

**Reference:** de Souza RJ, Mente A, Maroleanu A, Cozma AI, Ha V, Kishibe T, et al. Intake of saturated and trans unsaturated fatty acids and risk of all-cause mortality, cardiovascular disease, and type 2 diabetes: systematic review and meta-analysis of observational studies. BMJ. (2015) 351:h3978. doi: 10.1136/bmj.h3978

| **№ of studies** | **Risk of bias** | **Inconsistency** | **Indirectness** | **Imprecision** | **Others** | **Certainty** |  |
| --- | --- | --- | --- | --- | --- | --- | --- |
|  |  |  |  |  |  |  |  |
| **Coronary heart disease events (follow-up: range 1 year to 20 years)** | | | | | | |  |
| 12 | not serious | serious^a^ | not serious | serious^b^ | none | ⨁◯◯◯ |  |
|  |  |  |  |  |  | Very low |  |
| **Ischemic stroke (follow-up: range 1.5 years to 8.6 years)** | | | | | | |  |
| 12 | serious^c^ | serious^d^ | not serious | serious^e^ | none | ⨁◯◯◯ |  |
|  |  |  |  |  |  | Very low |  |

**Explanations**

^a^ *I^2^*=47%; P_het_=0.02; 8 studies had point estimates >1.0 and 9 had point estimates <1.0

^b^ Optimal information size met (n=6,383 events); summary RR crosses 1.0, however both bounds of 95% >0.8 and <1.2

^c^ Possibility of residual confounding always must be considered in observational studies. Newcastle-Ottawa score for 12 studies ranged from 5 to 8 (median=7). Main study limitations included incomplete adjustment for confounders (most commonly family history, and socioeconomic status), and failure to document losses to follow-up, and unclear outcome validation.

^d^ *I^2^*=59%; P_het_=0.002; 8 studies had point estimates >1.0 and 7 had point estimates <1.0.

^e^ Optimal information size met (n=6,226 events); summary RR crosses 1.0, however both bounds of 95% >0.8 and <1.2.

**Supplementary Table 10**.**Summary of excluded outcomes due to overlap.**

| **Author (year)** | **Outcomes** | **Population** | **Type of exposure** | **Comparator** | **Study follow-up range (years)** | **No. of**  **included**  **studies** | **Intervention****/comparator** | **Measures of effect** | **Effect size**  **(95%CI)** | **I^2^,%** | **AMSTAR-2 rating** | **GRADE rating** |
| --- | --- | --- | --- | --- | --- | --- | --- | --- | --- | --- | --- | --- |
| Mortality | | | | | | | | | | | | |
| Kim (2021) | All-cause mortality | Adults(>20 years) without pre-existing disease at baseline | Higher SFA intake: Higher intake category  g/day (15.5)  % total energy (range: 2.5% to 8.7%) | Reduced SFA intake:  Lowest intake category  g/day (24.3)  % total energy (range: 7.3% to 17.9%) | 6 to 26 | 11 | NR | RR | 1.03  (0.94 - 1.13) | 90.4 | Low | NR |
| de Souza (2015) | All-cause mortality | >16 years | Higher SFA intake: Higher intake category  g/day (13.7)  % total energy (range: 3% to 12.7%)  % total fat (12.7%) | Reduced SFA intake:  Lowest intake category  g/day (41)  % total energy (range: 7.3% to 21.3%)  % total fat (21.3%) | 6.6 to 19.3 | 5 | NR | RR | 0.99  (0.91 - 1.09) | 33 | Low | Very low |
| de Souza (2015) | Cardiovascular disease mortality | >35 years | Higher SFA intake: Higher intake category  g/day (7)  % total energy (range: 3% to 12.7%) | Reduced SFA intake:  Lowest intake category  g/day (41)  % total energy (range: 7.3% to 21.3%) | 6.6 to 19.3 | 3 | NR | RR | 0.97  (0.84 - 1.12) | 19 | Low | Very low |
| Mazidi (2020) | Cardiovascular disease mortality | >20 years | Higher SFA intake: According to percentiles, gr/day, % energy, increase in different units | Reduced SFA intake: According to percentiles, gr/day, % energy, increase in different units | 6.6 to 19.3 | 9 | NR | HR | 0.97  (0.84 - 1.11) | 30 | Critically low | NR |
| de Souza (2015) | Coronary heart disease mortality | >16 years | Higher SFA intake: Higher intake category  g/day (range: 13.7 to 34.7)  % total energy (range: 0.7% to 7.2%) | Reduced SFA intake:  Lowest intake category  g/day (range: 41 to 67.5)  % total energy (range: 1.5% to 14.8%) | 6 to 23 | 11 | NR | RR | 1.15  (0.97 - 1.36) | 70 | Low | Very low |
| Harcombe (2016) | Coronary heart disease mortality | 30 - 79 years | Higher SFA intake: Higher intake category | Reduced SFA intake: Lowest intake category | 6 to 20 | 6 | NR | RR | 1.08  (0.94 - 1.25) | 78.4 | Critically low | NR |
| Mazidi (2020) | Stroke mortality | >20 years | Higher SFA intake: According to percentiles, gr/day, % energy, increase in different units | Reduced SFA intake: According to percentiles, gr/day, % energy, increase in different units | NR | 3 | NR | HR | 1.03  (0.85 - 1.26) | 41 | Critically low | NR |
| Stroke | | | | | | | | | | | | |
| Kang (2020) | Stroke | 20 - 89 years | Higher SFA intake: Higher intake category (range: 15.4 to 50.4 gr/day) | Reduced SFA intake: Lowest intake category (range: 5.2 to 26.8 gr/day) | 7.4 to 20 | 14 ^a^ | NR | RR | 0.87 (0.78 - 0.96) | 37.8 | Critically low | NR |
| Cheng (2016) | Ischemic stroke | 20 - 89 years | Higher SFA intake: Higher intake category (range: 15.4 to 36 gr/day) | Reduced SFA intake: Lowest intake category (range: 7 to 20 gr/day) | 7.6 to 23 | 10 ^b^ | NR | RR | 0.90 (0.82 - 0.99) | 35.9 | Critically low | NR |
| Kang (2020) | Ischemic stroke | 20 - 89 years | Higher SFA intake: Higher intake category (range: 15.4 to 50.4 gr/day) | Reduced SFA intake: Lowest intake category (range: 5.2 to 26.8 gr/day) | 7.6 to 20 | 11 | NR | RR | 0.92 (0.82 - 1.03) | 30.7 | Low | NR |
| Muto (2018) | Ischemic stroke | >=34 years | Higher SFA intake: Higher intake category | Reduced SFA intake: Lowest intake category | 7.6 to 20 | 11 ^c^ | NR | HR | 0.89 (0.82 - 0.96) | 38.9 | Critically low | NR |
| Cheng (2016) | Hemorrhagic stroke | 20 - 89 years | Higher SFA intake: Higher intake category (range: 15.4 to 36 gr/day) | Reduced SFA intake: Lowest intake category (range: 7 to 20 gr/day) | 7.6 to 23 | 6 ^d^ | NR | RR | 0.76 (0.63 - 0.93) | 42.5 | Critically low | NR |
| Kang (2020) | Intracranial hemorrhage | 34 - 79 years | Higher SFA intake: Higher intake category (range: 15.4 to 50.4 gr/day) | Reduced SFA intake: Lowest intake category (range: 5.2 to 26.8 gr/day) | 11.1 to 14.3 | 4 ^e^ | NR | RR | 0.55 (0.41 - 0.73) | 0 | Low | NR |
| Incidence of cancer | | | | | | | | | | | | |
| Qiu (2016) | Ovarian Cancer | 25-69 years | Higher SFA intake: Higher intake category | Reduced SFA intake: Lowest intake category | 8.1 to 16.3 | 6 | NR | RR | 1.06 (0.89 - 1.26) | 44.37 | Critically low | NR |
| Diabetes | | | | | | | | | | | | |
| Neuenschwander (2020) | Type 2 diabetes | >30 years | Higher SFA intake: Higher intake category (range: 25.8 a 40.01 gr/day) | Reduced SFA intake: Lowest intake category (range: 12.37 to 33.3 gr/day) | 4.1 to 18 | 11 | NR | RR | 0.97  (0.92 – 1.02) | 34 | Low | Low |
| de Souza (2015) | Type 2 diabetes | > 34 years | Higher SFA intake: Higher intake category  g/day (range: 13.8 to 25.8)  % total energy (range: 7.6% to 36.1%) | Reduced SFA intake:  Lowest intake category  g/day (range: 25.8 to 86.6)  % total energy (range: 14% to 44.8%) | 5 to 14 | 8 | NR | RR | 0.95  (0.88 – 1.03) | 0 | Low | Very low |

**Abbreviations: No:** Number**; CI:** Confidence intervals**; I^2^:** Statistic assessment of heterogeneity**; %:** Percentage; **AMSTAR-2:** A Measurement Tool to Assess Systematic Reviews**; GRADE:** Grading of Recommendations, Assessment, Development, and Evaluations**; SFA:** Saturated fatty acids; **gr:** Gramos; **NR:** No reported**; RR:** Relative risk; **HR:** Hazard ratio**.**

1. Adjustment for confounding variables: Age, race, sex, systolic/diastolic blood pressure, number of cigarettes smoked, glucose intolerance, left ventricular hypertrophy, BMI, intake of energy, fruits, and vegetables, alcohol, atrial fibrillation, intake of a specific type of lipid, menopausal status, postmenopausal hormone use, vigorous exercise, usual aspirin use, multivitamin use, vitamin E use, n-3 fatty acid intake, calcium intake, hypertension, diabetes, high cholesterol levels, total energy intake, consumption of alcohol, potassium, fiber, fruits and vegetables, ose, city, BMI, mental stress,walking, sports, educational level, adult social class, marital status, fasting glucose,season, education,antihypertensive treatment, antihyperlipidemic treatment, leisure time,family history of myocardial infarction, on, income, smoking,coronary heart disease, walking and standing time, perceived mental stress, urban/rural, waist-to-hip ratio
2. Adjustment for confounding variables: No reported.
3. Adjustment for confounding variables: Age, sex, family history of myocardial infarction, menopausal status, diastolic blood pressure, atrial fibrillation, radiation dose, city, race, education, family income, aspirin use, sports during leisure time, walking and standing times, perceived mental stress, energy adjusted dietary intakes of carbohydrate, multivitamin use, vitamin E use, n-3 fatty acid intake, calcium intake, high cholesterol levels, smoking, alcohol, history of hypertension,  fruits and vegetables intake, glucose intolerance, physical activity, hormone replacement therapy use, total MET-hours per week, history of coronary disease, history of diabetes, aspirin use, use of antihyperten-sive medication, use of cholesterol-lowering medication, BMI, systolic blood pressure, total energy intake, protein, total fat and dietary fiber intake, left ventricular hypertrophy.
4. Adjustment for confounding variables: No reported.
5. Adjustment for confounding variables: Age, smoking, BMI, alcohol intake, menopausal status, postmenopausal hormone use, vigorous exercise, usual aspirin use, multivitamin use, vitamin E use, n-3 fatty acid intake, calcium intake, hypertension, diabetes, high cholesterol levels, total energy intake, sex, serum total cholesterol, mental stress, walking, sports, educational level, intake of vegetables, fruits, animal protein and calcium, walking and standing time, perceived mental stress.
